# Multivariate patterns of brain functional connectome associated with COVID-19-related negative affect symptoms

**DOI:** 10.1038/s41398-024-02741-1

**Published:** 2024-01-22

**Authors:** Nanfang Pan, Song Wang, Huan Lan, Xun Zhang, Kun Qin, Graham J. Kemp, Xueling Suo, Qiyong Gong

**Affiliations:** 1 Huaxi MR Research Center (HMRRC), Department of Radiology, West China Hospital of Sichuan University, Chengdu, China; 2 Research Unit of Psychoradiology, Chinese Academy of Medical Sciences, Chengdu, China; 3 Functional and Molecular Imaging Key Laboratory of Sichuan Province, Chengdu, China; 4 Department of Psychiatry, University of Cincinnati, Cincinnati, OH USA; 5 Liverpool Magnetic Resonance Imaging Centre (LiMRIC) and Institute of Life Course and Medical Sciences, University of Liverpool, Liverpool, UK; 6 Department of Radiology, West China Xiamen Hospital of Sichuan University, Xiamen, China

**Keywords:** Predictive markers, Human behaviour

## Abstract

Severe mental health problems with the representation of negative affect symptoms (NAS) have been increasingly reported during the coronavirus disease 2019 (COVID-19) pandemic. This study aimed to explore the multivariate patterns of brain functional connectome predicting COVID-19-related NAS. This cohort study encompassed a group of university students to undergo neuroimaging scans before the pandemic, and we re-contacted participants for 1-year follow-up COVID-related NAS evaluations during the pandemic. Regularized canonical correlation analysis was used to identify connectome-based dimensions of NAS to compute pairs of canonical variates. The predictive ability of identified functional connectome to NAS dimensional scores was examined with a nested cross-validation. Two dimensions (i.e. *mode stress* and *mode anxiety*) were related to distinct patterns of brain functional connectome (*r*^*2*^ = 0.911, *P*_*FDR*_ = 0.048; *r*^*2*^ = 0.901, *P*_*FDR*_ = 0.037, respectively). *Mode anxiety* was characterized by high loadings in connectivity between affective network (AFN) and visual network (VN), while connectivity of the default mode network with dorsal attention network (DAN) were remarkably prominent in *mode stress*. Connectivity patterns within the DAN and between DAN and VN, ventral attention network, and AFN was common for both dimensions. The identified functional connectome can reliably predict *mode stress* (*r* = 0.37, MAE = 5.1, *p* < 0.001) and *mode anxiety* (*r* = 0.28, MAE = 5.4, *p* = 0.005) in the cross-validation. Our findings provide new insight into multivariate dimensions of COVID-related NAS, which may have implications for developing network-based biomarkers in psychological interventions for vulnerable individuals in the pandemic.

## Introduction

Since the World Health Organization declared coronavirus disease 2019 (COVID-19) an International Public Health Emergency on 30th January 2020 [1], the pandemic brought various physical health detriments with huge socioeconomic impact [2, 3]. In addition, there is increasing evidence that the pandemic and its accompanying confinement measures (e.g. social distancing and quarantine) lead to serious and sustained mental health problems [4]. Particularly, individuals facing such invisible, uncertain and continuous pandemic threats are more susceptible to negative affect symptoms (NAS) such as stress, anxiety, depression, burnout, and psychological trauma [5, 6]. Ways to identify individuals who are at increased risk of NAS are urgently needed to target preventive interventions and long-term treatment. It is worth noting that the NAS and psychiatric symptoms are more severe among college students in relative to other populations [7, 8], given that they may rely more on the internet and social media for COVID-linked information [9, 10]. Therefore, in the research reported here, we explored the pre-pandemic brain functional connectome of a sample of university students for features predictive of patterns of NAS during the post-peak period of the pandemic.

The evolving subspeciality of psychoradiology offers a way to study links between the brain and mental health [11, 12]. Based on this brain-behavior investigation, recent studies have identified brain functional and structural features associated with dimensions of NAS including anxiety, stress, fear, depression and posttraumatic stress symptoms in both COVID-19 survivors and general populations [13–16]. However, studies focusing on single behavioral measures or symptom domains, fail (as in the parable of the blind men and the elephant [17]) to take full account of the interdependence of behavioral and symptom variables [18], which limits their explanatory power to grasp the full picture of the brain-NAS associations [19]. Even when factor analysis is used to examine the latent dimensions of behavioral characteristics [20, 21], this typically relies on the covariance structure of behavioral data. Multivariate data-driven analyses based on multi-dimensional brain and behavioral data offer a way to escape these limitations to build up generalizable brain–behavior associations [22]. One such is canonical correlation analysis (CCA), which is designed to model mutual dependencies between behavioral measures and brain features [23]. CCA identifies the complex linear relationships of many-to-many associations between two-dimensional datasets to focus on patterns of brain features rather than features in isolation [18, 19, 24, 25].

In this study, therefore, we sought to delineate the multivariate patterns of brain functional network abnormalities associated with a broad array of COVID-19-related NAS, using pre-pandemic resting-state functional MRI (rs-fMRI) data and 17 during-pandemic assessments of NAS on a cohort of university students. The functional brain connectome was first modeled, and regularized CCA was then used to discover distinct patterns of covariation between functional connectome properties and COVID-19-related NAS. Cross-validation analyses based on a machine learning pipeline evaluated the predictive ability of the identified functional connectome properties towards dimensional scores of NAS [26].

## Materials and methods

**Fig. 1:**
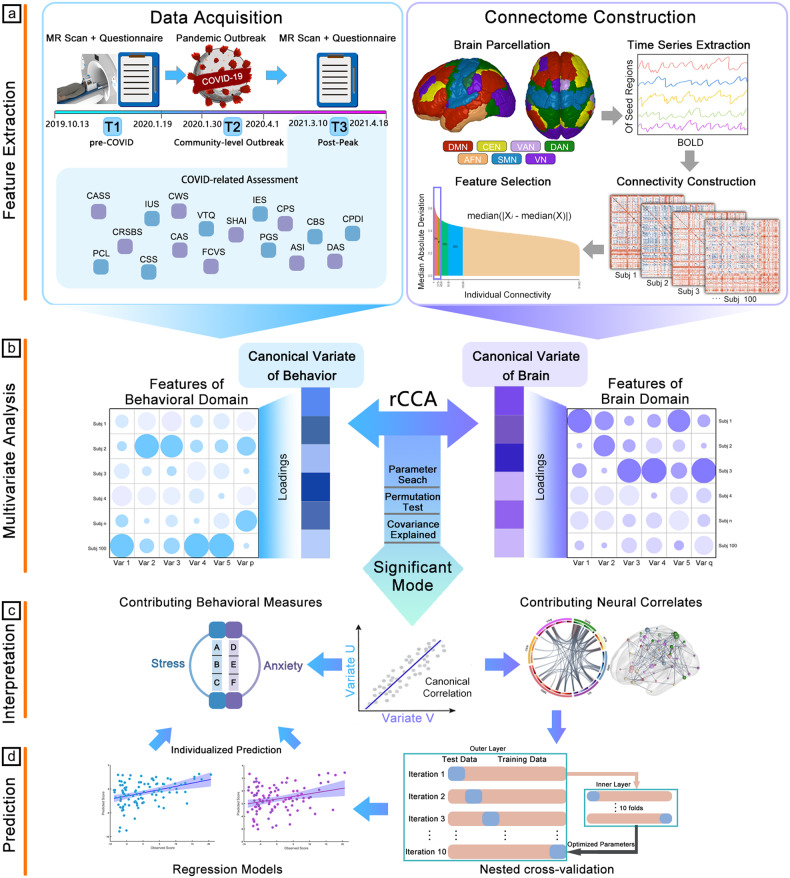
Flowchart of data acquisition and analysis by Regularized Canonical Correlation Analysis (rCCA). In (**a**), the data acquisition panel shows the timeline of sample enrollment, the main events of the COVID-19 pandemic in China, and the psychological measurements; the connectome construction panel shows the process from building the brain connectivity matrix to feature selection. **b** The process of multivariate analysis, in which rCCA is used to capture the maximized common variations for brain and behavioral data. **c** Interpretation in terms of neural mechanisms, using the brain and behavioral features that stably contribute to each covariation mode. **d** The machine learning pipeline established to predict the scores of psychological traits based on the brain dataset, and the examination of potential confounding effects of age and sex. Abbreviations: CPDI COVID-19 Peritraumatic Distress Index, CASS COVID-19 Anxiety Syndrome Scale, CRSBS Coronavirus Reassurance-Seeking Behaviors Scale, CAS Coronavirus Anxiety Scale, CBS COVID-19 Burnout Scale, CWS COVID-19 Worry Scale, SHAI Short Version of Health Anxiety Inventory, PGS Pandemic Grief Scale, FCVS Fear of COVID-19 Scale, CPS COVID-19 Phobia Scale, CSS COVID-19 Stress Scales, VTQ Vicarious Traumatization Questionnaire, IUS Intolerance of Uncertainty Scale, ASI Anxiety Sensitivity Index, DAS Death Anxiety Scale, PCL PTSD Checklist For DSM-5, IES Revised Version of the Impact of Event Scale, DMN Default Mode Network, CEN Central Executive Network, DAN Dorsal Attention Network, AFN Affective Network, VN Visual Network, VAN Ventral Attention Network.

An overview of data collection and analytical procedures is shown in Fig. 1.

### Data collection

#### Participants

Before the pandemic (from 13th October 2019 to 19th January 2020), we recruited 151 general college students with no known history of psychiatric or neurological disease to complete multimodal neuroimaging scanning. All were re-contacted for follow-up psychological evaluations during the post-peak period of the pandemic (from 10th March to 18th April 2021) [26], and 127 responded and finished evaluation procedures. None of the participants were infected with COVID-19 prior to their follow-up assessments, as evidenced by the examination of their Health Code, which documented both current and historical infection information [27, 28]. Bogus items were employed to identify any not paying attention or responding dishonestly [29], and 12 subjects were excluded. This study was approved by the local research ethics committee of West China Hospital. All subjects gave written informed consent before participation.

#### MRI protocol procedure

All subjects underwent MRI scanning using a Siemens (Erlangen, Germany) Trio 3.0 Tesla system with a 12-channel head coil. Structural MRI and rs-fMRI data were collected as detailed in Supplementary Methods.

#### Psychological assessment

Psychological evaluations were performed with 17 self-reported questionnaires/scales, including items on COVID-19-related NAS such as depression, anxiety, stress, burnout, and psychological trauma, as detailed in Supplementary Methods.

### Neuroimaging data preprocessing

The rs-fMRI data preprocessing was conducted using fMRIPrep v20.2.6 based on Nipype v1.7.0 in the following steps [30, 31]: skull strip, slice timing correction and susceptibility distortion correction using 3dTshift from AFNI, intrasubject boundary-based registration using FLIRT from FSL, spatial normalization using ANTs, and estimation of nuisance confounders (for details of the fMRIPrep pipeline, see https://fmriprep.org/). The functional reference was volumetrically resampled into standard MNI space [32]. Confounding components were calculated including anatomical and temporal component-based noise correction (CompCor) variance, DVARS, motion parameters, framewise displacement (FD) and detected outliers to regress out physiological and movement noise [33, 34]. Motion parameters in terms of the reference are evaluated using MCFLIRT, and frames exceeding 0.5 mm FD or 1.5 standardized DVARS were annotated as motion outliers. After fMRIPrep preprocessing, we applied removal of the first 10 images and spatial smoothing with a 6 mm full-width half-maximum. Confounding components of scrubbing indicators, motion regressors, and white matter and cerebrospinal fluid physiological signals were regressed out with the band-pass temporal filter at 0.008-0.09 Hz [35]. We excluded 15 participants with mean FD > 0.25 mm as motion outliers [36], and 100 participants (58 females, mean age = 22.4 ± 2.1 years) remained for further analyses.

### Brain connectome construction and feature selection

To build the brain functional connectivity matrix, we selected cortical seeds based on a 100-area parcellation and subcortical seeds based on 36 subregions in the human Brainnetome atlas [37, 38], which has been widely used in neuroimaging studies [26, 39]. To enhance interpretability of network findings, the parcellation areas were assigned to 7 predefined networks: the default mode network (DMN), central executive network (CEN), ventral attention network (VAN), dorsal attention network (DAN), affective network (AFN), sensorimotor network (SMN) and visual network (VN) (Supplementary Fig. S1 and Table S1) [40]. We computed Pearson’s correlations of the mean time series of all pairs of regions, transforming correlation coefficients to z-values for the normality. To minimize effects of confounding factors, we controlled throughout for age, sex, and mean FD. For each individual, we obtained a 136*136 symmetric connectome matrix with 9180 connectivity features.

To preserve the most predictive connectivity features for NAS, those with excessive variance across individuals [i.e. the top connectivity pairs with the highest median absolute deviation (MAD)] would remain as the essence of the neuroimaging data to achieve dimensionality reduction and keep the maximum robustness and resilience to outliers [18, 41]. We presented the connectome matrix with various MAD thresholds (Supplementary Fig. S2), and selected the top 5% links (459 connections) that varied most across individuals for further analyses to enhance both the variability and interpretability. The total scores of each questionnaire (17 measures) became the behavioral input into the modeling [42].

### Regularized canonical correlation analysis

Since mass univariate testing in model-building generally fails to establish generalizable brain–behavior covariations [43], multivariate analysis is the best way to explore relationships between brain and behavioral datasets in a data-driven way. We used the regularized CCA (rCCA) method in the mixOmics R package [44], which is well-validated in biopsychological studies and especially suitable where there are more variables than samples [45], to build up maximal correlations between linear combinations of both behavioral and brain connectome data and to compute pairs of canonical variates [42].

We introduced the regularization approach to address the issue of high dimensionality and collinearities, adding ridge penalties (λ_1_, λ_2_) to the diagonal of brain and behavioral variables, respectively, to make them invertible [46]. We evaluated and optimized various pairs of regularization parameters by grid search in the constrained range 0.01–0.99 (length = 20) to yield the highest canonical correlation of the first variate. To avoid over-fitting and false-positive inflation, we conducted 5000 permutation tests by shuffling the rows of brain connectome data, and created a null distribution of canonical correlations to determine significant covariation modes [47]. The *p* value was computed based on the number of permutating canonical correlations that exceeded the performance of original rows, and we reported covariation modes that were statistically significant at false discovery rate correction (FDR, *q* < 0.05) and which explain more than 5% of the covariance for further analyses [48].

### Covariation mode interpretation

Identifying covariation modes by rCCA allows us to interpret the multivariate relations between brain connectome and behavioral features in a low-dimensional space. To determine features that stably contribute to each covariation mode, we computed Pearson correlation coefficients (i.e. canonical loadings) between each canonical variate and its original variables [24, 48]. The most highly weighted features for each mode may provide a primary indication for its essence.

To help understand the brain connectome features which are highly correlated with identified covariation modes, these connectivity loadings were assigned to within- and between-network communities in terms of 7 network parcellations of Yeo’s and loadings of each were summed as measures of network strength based on these community assignment [26, 40]. Summed loadings for each seed region were then calculated as a measure of node strength, reflecting its ability to facilitate functional interaction within the connectome [49].

### Pattern prediction

NeuroMiner (www.pronia.eu/neurominer/) was used to establish a machine learning pipeline to predict the dimensional scores of behavioral measures based on the brain dataset [50]. For unbiased estimation of generalizability, and to avoid information leakage in the training process, we performed nested cross-validation with a 10-fold cycle in both the outer and inner layer, and repeated cross-validation at the outer layer by 10 random permutations [51, 52]. The best performing models with parameters obtained from the inner layer would be applied to the data of the outer layer. Input features were links of identified modes that consistently contributed, and the targets were the total standardized scores of identified behavioral measures [53]. Brain features were employed covarying age, sex, and mean FD and scaling from 0 to 1. To further reduce the dimensionality, those were projected to eigenvariate space contributing to retaining 80% of the variance [52]. We used a linear support vector machine (SVM) to undertake a greedy forward search for optimized parameters to create fitting models that optimally predict the observed scores [54]. Permutation tests (1000 times on label) were used to inform whether our predictive model was significantly different from those with ensemble predictors (*p* < 0.01) [55].

### Confounding effects on connectome-behavioral correlations

We repeated the rCCA analysis without covarying age and sex for features [18], and established a generalized linear model to evaluate the possibility of a linear relationship between age and identified brain connectivity, and of differences in brain patterns between males and females. These evaluations were performed for each covariation mode.

## Results

### Psychological evaluations and covariation mode identification

**Fig. 2:**
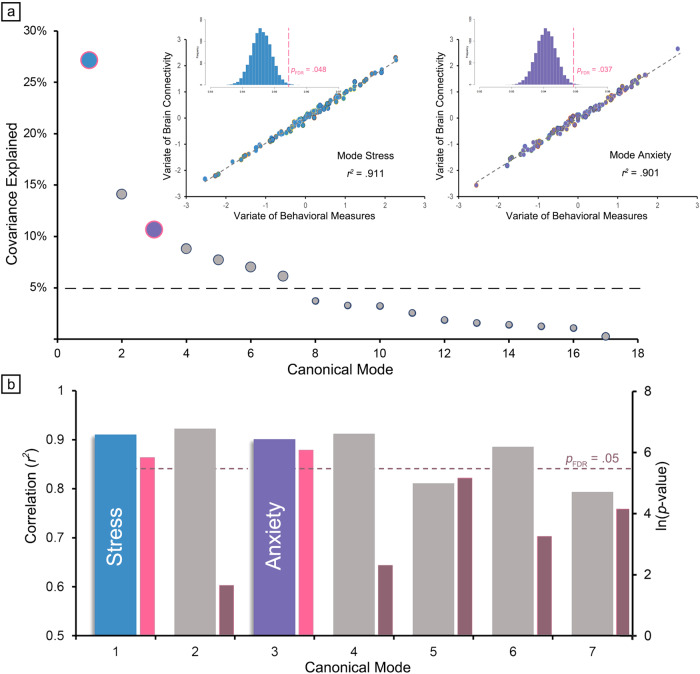
Multivariate patterns of covariation mode. **a** Shows how we extracted the first seven covariation modes that exceeded the predefined threshold of covariance explained (dashed line). Scatter plots exhibit the correlated multivariate patterns of brain connectivity and psychological measures, and the histograms display the null distribution of permutating rCCA combinations. **b** Shows how two covariation modes were statistically significant by permutation testing with FDR correction (*p*_FDR_ < 0.05).

**Fig. 3:**
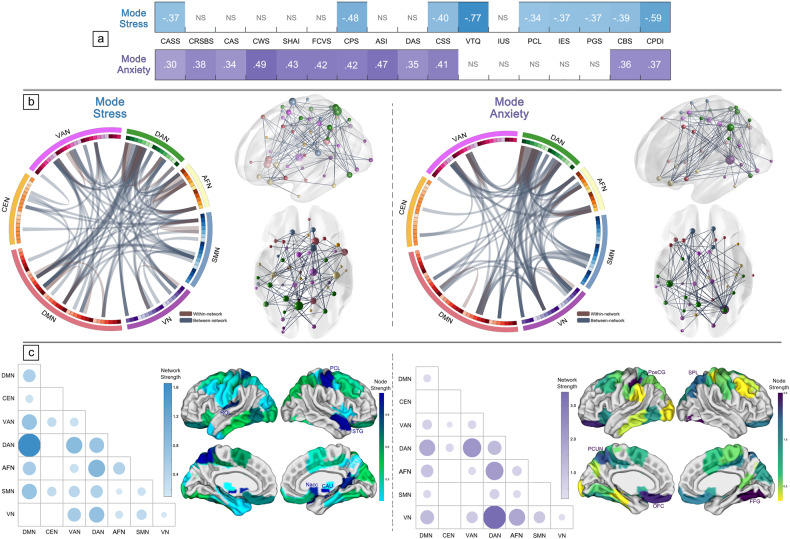
Data-driven patterns of behavioral measures and brain connectivity contributing to identified covariation modes. **a** The prevalence of each psychological measure in the covariation modes: *mode stress* and *mode anxiety*. **b** The identified neural connectivity patterns with edges that stably contributed to each of these modes, delineated by Circos plots and ball-and-stick brain diagrams. **c** The identified brain connectome patterns at the network community level and nodal level, with the neuroanatomical distribution. ROL Rolandic operculum, PCL paracentral lobule, STG superior temporal gyrus, Nacc nucleus accumbens, CAU caudate, PosCG postcentral gyrus, SPL superior parietal lobule, PCUN precuneus, OFC orbitofrontal cortex, FFG fusiform gyrus.

The means, standard deviations, ranges and bivariate correlations of the pandemic-specific psychological evaluations are shown in Supplementary Table S2. We employed rCCA as a data-driven approach to unfold multivariate correlations between brain connectome and psychological measures. Following the grid search of optimized regularization parameters of rCCA (Supplementary Fig. S3) we found that 7 covariation modes exceeded the predefined threshold of covariance explained (Fig. 2a). After assessing the significance of each by permutation testing, we selected the first and third of those seven covariation modes (*r*^*2*^ = 0.911, *P*_*FDR*_ = 0.048; *r*^*2*^ = 0.901, *P*_*FDR*_ = 0.037, respectively, Fig. 2b). As the specific pattern of each of these two covariation modes anchored on weighted features of two input datasets, we labeled them *mode stress* and *mode anxiety* respectively, based on the selection of their contributing behavioral features (9/17 measures consistently contributed to *mode stress* and 12/17 measures to *mode anxiety*; see Fig. 3a).

### Functional connectomes linked to stress and anxiety covariation modes

To interpret the brain connectome driving the multivariate relationships of each mode, we attributed those highly weighted connectivity pairs to 7 network communities (Fig. 3b, c). Most within-network links were assigned to DAN for both modes (network strength = 0.71 and 1.24, respectively). *Mode stress* was characterized by highest loadings in connectivities between DAN and DMN among between-network pairs (network strength = 1.66), while connectivities between DAN and VN were remarkably prominent in *mode anxiety* (network strength = 3.40). Considering the neuroanatomical distribution and canonical loadings of all seed regions, the top three regions with the highest node strength in *mode stress* were the paracentral lobule, caudate and temporal pole, and those in *mode anxiety* were the fusiform, postcentral gyrus, and orbitofrontal cortex (Fig. 3c and Supplementary Table S3).

**Fig. 4:**
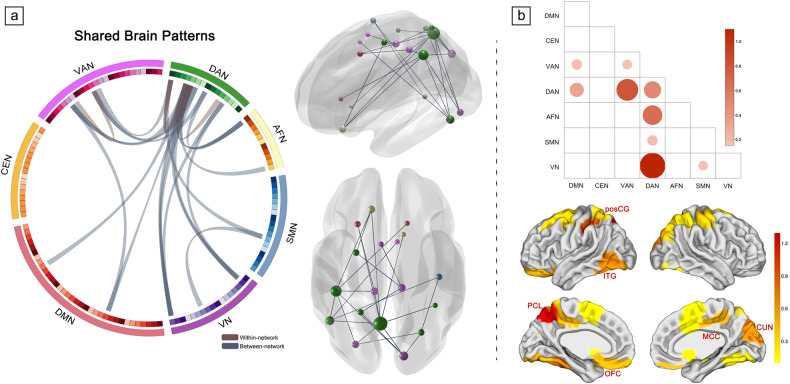
Desegregated brain patterns as overlaps across two modes. **a** Neural connectivity patterns with shared edges that stably contributed to both modes. **b** Overlapping brain connectome patterns at the network community level and nodal level, with the neuroanatomical distribution. PosCG postcentral gyrus, ITG inferior temporal gyrus, PCL paracentral lobule, OFC orbitofrontal cortex, MCC middle cingulate cortex, CUN cuneus.

When mapping connectome of each other, 25 pairs of brain connectivity overlapped across both modes (Fig. 4a). In particular, alterations of connectivity from DAN to VN, VAN, and AFN (mean network strength = 1.10, 0.80, and 0.65 respectively; Fig. 4b) accounted for most of the shared pattern. Regions mainly contributing to both stress and anxiety patterns were the paracentral lobule, postcentral gyrus and inferior temporal gyrus as hub nodes in the connectome (Fig. 4c). For mode-specific connectivity patterns, DAN-DMN connectivity predominated in *mode stress*, and connectivity from VN to AFN largely constituted in *mode anxiety* (see Supplementary Fig. S4).

### Individualized prediction of mode stress and anxiety

**Fig. 5:**
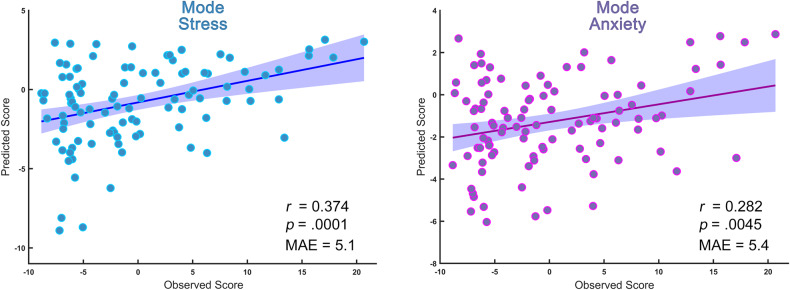
Prediction performance of identified brain connectome for scores of the two modes. Machine learning pipeline based on nested cross-validation established the predictive models, and the scatter plots of linear association between observed and predicted scores is shown (*mode stress* left, *mode anxiety* right), with *r* and mean absolute error (MAE) values.

We conducted 10-fold nested cross-validation with a linear SVM using predictors trained on identified links of modes. Age, sex, and mean FD were modeled with linear regression models as covariates. Optimized parameters for the performing models are shown in Supplementary Fig. S5. With these individualized models, we achieved robust predictions of *mode stress* (*r*_(predicted, observed)_ = 0.37, mean absolute error (MAE) = 5.1, *p* < 0.001) and *mode anxiety* (*r*_(predicted, observed)_ = 0.28, MAE = 5.4, *p* = 0.005, Fig. 5). Their predictive performance was significant, informed by the permutation test (both *p* < 0.001, Supplementary Fig. S6).

### Age effects and sex differences

Covariation modes derived from the rCCA pipeline with non-regressed features were closely correlated with the covariate-regressed ones (*mode stress*: *r*_connectivity_ = 0.960, *r*_psychological_ = 0.961; *mode anxiety*: *r*_connectivity_ = 0.965, *r*_psychological_ = 0.966). Linear relations between age and brain patterns were not significant in either covariation mode (both *P*_FDR_ > 0.05). Brain connectivity associations with both modes were stronger in females than males (brain connectivity score: *mode stress* 0.011 vs. −0.016; *mode anxiety* 0.012 vs. −0.017; Supplementary Fig. S7), but the differences were not significant (both *P*_FDR_ > 0.05).

## Discussion

Multivariate data-driven analyses identified 2 covariation modes (*mode anxiety* and *mode stress*) between the brain functional connectome and pandemic-specific NAS measures in a cohort of college students. Connectivity pairs within DAN and pairs of DAN with VAN, VN, and AFN were common to both modes. *Mode anxiet*y was characterized by connectivities of AFN with VN, *mode stress* by connectivity pairs of DMN with DAN. These two connectome-guided dimensions of COVID-19-related NAS might serve as a foundation for developing network-based biomarkers of NAS-linked mental disorders.

Notably, these brain-driven modes (*stress* and *anxiety*) incorporated NAS across multi-dimensional psychological characteristics consistent with common psychological reactions to the COVID-19 pandemic [56, 57]. *Mode stress* comprised psychological constructs including stress syndrome, burnout, dysfunctional grief, vicarious traumatization, posttraumatic stress symptoms, the impact of traumatic events, and general distress. In contrast, *mode anxiety* represents various types of anxiety: coronavirus anxiety syndrome, coronavirus reassurance-seeking behaviors, coronavirus anxiety, coronavirus worry, coronavirus fear, coronavirus phobia, general health anxiety, anxiety sensitivity index, and death anxiety. Notably, several constructs (anxiety syndrome, phobia, stress syndrome, burnout and general distress) were simultaneously loaded on both modes, suggesting that these may have different neurobiological underpinnings [18]. Rather than averaging over many NAS within a dimension, the rCCA method selects the specific items most tightly linked to patterns of connectivity. While the pandemic has led to a range of enduring mental health effects among vulnerable populations and the general public, with a particular impact on college students [58–60], this study underscores anxiety and stress as the primary potential challenges for public mental health.

As noted, while each of these covariation modes was associated with a unique connectome pattern, common to both were altered functional connectivities within DAN, and between DAN and VAN, VN, and AFN. We suggest that these reflect a common neurobiological mechanism underlying vulnerability to a wide range of COVID-19-related NAS. The DAN is primarily involved in allocating attention [61], and compromised executive functions supported by this network may contribute to the abnormalities of focus of attention and concentration [62]; dysconnectivity within DAN has been linked to other cognitive-affective mental disorders [63–66]. In contrast to the DAN’s dominant role in top-down voluntary attention to salient stimuli, the VAN is mainly responsible for mediating involuntary salient external and internal stimuli [61]. It has been suggested that bottom-up signal transfer from VAN to DAN impairs top-down attentional control [67]. Our findings that weakened DAN-VAN connectivity was related to anxiety and stress suggests a network mechanism whereby the decreased top-down flow from DAN impairs suppression of VAN activation, resulting in increased attention to the environment which may increase risk for attentional control deficits under the stress of the COVID-19 pandemic. Moreover, convergent evidence shows that DAN is richly interconnected with VN, and with multiple modules of visual processing, critical in emotion regulation [68]. Studies of posttraumatic stress disorders have found lower activity in visual and dorsal and ventral attention systems, suggesting that abnormal visual processing may be related to attentional dysfunction [69]. In major depression disorder, lower functional connectivity between DAN and VN have been linked to impaired ability to distract attention from negative stimuli [70]. Our findings of altered DAN-VN connectivity may reflect the modulation of visual properties of stimuli by higher-level information. In addition, AFN, located in the temporo-amygdala-orbitofrontal circuit, is involved in the integration of visceral sensation and emotion with semantic memory and related behavior; weaker connections to AFN may contribute to the negative behavioral strategies [71]. Studies of major depression disorders have found decreased functional connectivity between AFN and DAN, possibly related to mood fluctuation and inattention [72]. Our finding of altered DAN-AFN connectivity might be related to pandemic-induced emotional dysregulation.

In addition to these common characteristics present across dimensions, each dimension of NAS was associated with a unique, highly correlated connectome pattern. *Mode anxiety* was characterized by high loadings in connectivities between AFN and VN, while connectivities of the DMN with DAN were prominent in *mode stress*. The VN-AFN connectivity associated with *mode anxiety* may suggest a link between this network and emotion-related processes [73]. Activities in the affective/limbic (e.g. amygdala) and visual cortex closely co-vary during emotional picture viewing, increasing with rated picture arousal [74]; and fearful individuals reacting to specific fear cues show parallel activation in these brain regions compared with non-fearful controls, which may contribute to anxiety-associated psychopathology (e.g. generalized anxiety disorder) [75]. Processing of environmental fear/threat-related stimuli is modulated by the functional connectivity between the amygdala (belonging to AFN) and visual cortex, which may provide targets for interventions in anxiety-related disorders [76, 77]. On the other hand, the DMN is well-known for self-referential processing and autobiographical rumination [78] and its disruption has been related to impaired self-awareness [79], which can be depleted by stressful events [80]. The intrinsic activity and connectivity of DMN have been associated with the stress process [81]; functional abnormalities in the DMN are linked to stress-related mental health issues such as general distress and posttraumatic stress disorders [26, 82, 83]. The DAN could also play a role in emotional regulation, and in the higher state of vigilance and awareness, which is typical of stressed-induced hyperemotionality [84]. COVID-19 infection risk in particular is related to reduced DMN-DAN functional connectivity [85]. Our recent study also found that lower DMN-DAN connectivity predicted more severe long-standing distress symptoms during the pandemic. Stronger DMN-DAN connectivity is related to contextualizing emotional experience by reappraisal [68], by which individuals might mitigate distress due to pandemic stressors. Thus, individuals with weak DMN connections appear more likely to be subject to the various stresses of the COVID-19 pandemic.

This study has several limitations. First, although the analyses were based on prospective data they cannot prove causation, although we have suggested ways in which the reported associations may reflect biological, and likely causative, mechanisms [48]. Future longitudinal studies with MRI and behavioral data at multiple time points are needed to understand how the covariation between the brain functional connectome and NAS evolves. Second, we examined a rather limited and homogeneous group of college students, whose levels of NAS or psychiatric symptoms could be elevated [7, 86], making them less representative of other populations. Our findings need further validation using an independent and larger sample, and studies are warranted of other populations (e.g., general adults, children and the elderly) and highly vulnerable groups (e.g., frontline medical workers). Third, although group-level atlases have been widely used in constructing brain connectome [87], they may miss subtle brain–behavior associations in assessing individual-level functional data [88]. Finally, behavioral data in our study were derived from self-reported questionnaires, which are subjective and assess a mix of self-beliefs, values and attitudes [89]. Notably, there could be potential disparities between subjective and objective measurements in the context of COVID-19 pandemic [90, 91]. Therefore, more objective assessments (e.g., other-report evaluations and task-based measures) are warranted to mitigate the impact of response bias.

## Conclusion

This study is the first to reveal multivariate patterns of brain functional connectome encoding modes of COVID-19-related NAS in a sample of healthy university students. We identified 2 modes (*anxiety* and *stress*) that represent patterns of covariation between pre-pandemic brain networks and NAS manifestations during the pandemic. Connectivities within DAN and between DAN and VAN, VN, and AFN were common to both modes; *mode anxiety* was characterized by connectivities of AFN with VN, while connectivities of the DMN with DAN were prominent in *mode stress*. These may throw some light on the neurobiological basis of COVID-19-related NAS, with possible implications for developing psychological therapies and brain interventions for individuals at particular risk of psychological dysfunction and distress [92, 93].

### Supplementary information

Supplementary

## Data Availability

Those key scripts and data information for behavioral and neuroimaging analyses are available at https://osf.io/2k4ex/, and other data that support the findings of the present study are available from the corresponding author through reasonable request.
