# Supplementary material for: Multivariate patterns of brain functional connectome associated with COVID-19-related negative affect symptoms
Source: Transl Psychiatry. 2024 Jan 22;14:49. doi: 10.1038/s41398-024-02741-1 (PMC10803304; doi:10.1038/s41398-024-02741-1)
Supplement: Supplementary file 1 — Supplementary [file 41398_2024_2741_MOESM1_ESM.docx]

**Multivariate Patterns of Brain Functional Connectome Underlying COVID-related Negative Affect Symptoms**

**Online Supplement**

**Supplementary Methods**

**Supplementary Tables and Figures**

**Figure S1.** Brain Functional Network Parcellation

**Figure S2.** Feature Selection Based on Median Absolute Deviation

**Figure S3.** Grid Search for Regularization Parameters

**Figure S4.** Mode-specific Brain Connectivity Patterns

**Figure S5.** Parameter Search for Nested Cross-validation

**Figure S6.** Permutation Test for Prediction Models

**Figure S7.** Developmental Effects and Sex Differences

**Table S1.** Seed Regions of Brain Functional Parcellation

**Table S2.** Bivariate Correlations of Pandemic-specific Psychological Evaluations

**Table S3.** Identified brain connectome patterns at the nodal level

**Reference**

The supplementary material has been provided by the authors to give readers additional information about their work.

**Supplementary Methods**

1. MRI Protocol Procedure.

We used echo-planar imaging sequence to acquire the rs-fMRI data (TR = 2000 ms, TE = 30 ms, flip angle = 90°, 30 slices, voxel size = 3.75 × 3.75 × 5 mm^3^, field of view = 240 × 240 mm^2^, matrix = 64 × 64, 240 volumes). To achieve high data quality, participants with excessive head motion (transformation distance > 1 mm, rotation angle > 1°) were required to undergo rs-fMRI scanning again until they meet the criteria. High-resolution T1-weighted anatomical MRI images were obtained (TR = 1900 ms, TE = 2.26 ms, flip angle = 9°, 176 slices, voxel size = 1 × 1 × 1mm^3^, matrix size = 256 × 256). During the scanning, participants were instructed to remain relaxed, close their eyes, and stay awake without systematically thinking.

1. Psychological Assessments

For each participant, all psychological assessments in this section were completed after the MRI scanning. Participants were required to rate each item according to their actual situations and feelings during the past month. All tests were written in simplified Chinese through a Chinese online survey website (http://www.sojump.com) and a research assistant supervised the test session.

*Anxiety Sensitivity Index (ASI)*

Developed by Taylor et al. (2007) to measure anxiety-related cognitive (e.g., “When my mind goes blank, I worry there is something terribly wrong with me”), social (e.g., “I think it would be horrible for me to faint in public”) and physical (e.g., “When my throat feels tight, I worry that I could choke to death”) concerns, ASI is an 18-item questionnaire using a 5-point Likert scale ranging from 1 (very little) to 5 (very much). It yields a total score by summing the responses of all items and higher scores indicate higher levels of anxiety sensitivity. The Chinese version of ASI has been well-validated and applied in a body of studies and shown good psychometric properties in different populations (Cai et al., 2018; Han et al., 2020; Wang et al., 2014). The Cronbach's α of ASI in our sample was 0.93, indicating excellent internal reliability.

*Coronavirus Anxiety Scale (CAS)*

The CAS (Lee, 2020) is a unidimensional questionnaire that includes 5 items (e.g., “I had trouble falling or staying asleep because I was thinking about the coronavirus”), with each item rating on a 5-point Likert scale from 1 (not at all) to 5 (nearly every day). It yields a total score and higher scores indicate more COVID-specific anxiety symptoms. The Chinese version of CAS has shown satisfactory reliability and validity (Chen et al., 2021).The Cronbach's α of CAS in our sample was 0.95, indicating excellent internal reliability.

*Coronavirus Reassurance-Seeking Behaviors Scale (CRSBS)*

This survey (Lee et al., 2020) aims to capture an individual’s reassurance-seeking behaviors during the pandemic; it has 5 items (e.g., “I took my temperature to see if I was infected with the coronavirus disease”) that are loaded on a single factor structure. Participants are asked to rate how frequently they engaged in reassurance-seeking activities from 1 (not at all) to 5 (nearly every day). A total score of CRSBS can be obtained by summing the responses of all items and higher scores indicate more reassurance-seeking behaviors. The Chinese version of CRSBS was developed by investigators proficient both in Chinese and English using a standardized translation and back-translation procedure (Beaton et al., 2000). The Cronbach's α of CRSBS in our sample was 0.86, indicating satisfactory internal reliability.

*COVID-19 Anxiety Syndrome Scale (CASS)*

The 9-item CASS (Nikčević & Spada, 2020) tends to evaluate the presence of avoidance (e.g., “I have avoided using public transport because of the fear of contracting coronavirus (COVID-19)”) and perseverate thinking (e.g., “I have checked my family members and loved one for the signs of coronavirus (COVID-19)”) linked with the COVID-19 anxiety syndrome. Each item is rated on a 5-point Likert scale from (never) to 5 (always) and a total score can be yielded with higher scores indicating more anxiety symptoms. The Chinese version of CASS has been developed in previous research and demonstrated adequate reliability and validity (Xin et al., 2022). The Cronbach's α of CASS in our sample was 0.88, indicating satisfactory internal reliability.

*COVID-19 Burnout Scale (CBS)*

Adapted from the Burnout Measure-Short Version (Malach-Pines, 2005), the CBS (Yıldırım & Solmaz, 2022) is a unidimensional scale including 10 items (e.g., “When you think about COVID-19 overall, how often do you feel hopeless?”). Participants are asked to rate each item on a 5-point Likert scale ranging from 1 (never) to 5 (always). A total score of CRSBS can be computed by summing the responses across 10 items and higher scores represent higher COVID-19 burnout syndrome. The psychometric properties of the Chinese version of CBS have been confirmed and established in previous studies (Wang et al., 2022; Ye et al., 2022). The Cronbach's α of CBS in our sample was 0.91, indicating excellent internal reliability.

*COVID-19 Peritraumatic Distress Index (CPDI)*

Developed by Qiu et al. (2020) among Chinese general populations, the CPDI measures the frequency of COVID-reduced distress symptoms including depression, anxiety, specific phobias, physical symptoms, compulsive and avoidance behaviors, cognitive changes, and dysfunctional social competence, with an example item as “I feel tired and even exhausted at times”. This questionnaire consists of 24 items that are rated on a 5-point Likert scale ranging from 1 (never) to 5 (always). By summing participants’ responses on each item, a total score is obtained and higher scores represent more distress symptoms linking with COVID-19. The CPDI has been widely used in previous studies and has shown satisfied reliability and validity (Jiménez et al., 2021; Marzo et al., 2021; Shrestha et al., 2020). The Cronbach's α of CPDI in our sample was 0.93, indicating excellent internal reliability.

*COVID-19 Phobia Scale (CPS)*

Based on the DSM-V criteria for specific phobias, the CPS (Arpaci et al., 2020) purposes to assess the persistent and excessive fear of the COVID-19. It consists of 20 items that are rated on a 5-point Likert scale from 1 to 5. The CPS yields a 4-factor structure: psychological (e.g., “The pace that coronavirus has spread causes me great panic”), psycho-somatic (e.g., “I experience tremors due to the fear of coronavirus”), economic (e.g., “I stock food with the fear of coronavirus”), and social (e.g., “I am unable to curb my anxiety of catching coronavirus from others”). A total score can be calculated as the sum of responses across 20 items, with higher scores indicating more severe COVID-19 phobia. The Chinese version of CPS has been applied in different populations and has shown satisfied psychometric properties (Chu et al., 2022; Zhao et al., 2023). The Cronbach's α of CPS in our sample was 0.90, indicating excellent internal reliability.

*COVID-19 Stress Scales (CSS)*

Developed by Taylor et al. (2020) to measure COVID-related stress syndrome, the CSS consists of 36 items that are rated on a 5-point Likert scale from 1 (not at all) to 5 (extremely). It yields six dimensions: danger (e.g., “I am worried about catching the virus”), contamination (e.g., “I am worried about taking change in cash transactions”), socioeconomic consequences (e.g., “I am worried that grocery stores will close down”), xenophobia (e.g., “I am worried about coming into contact with foreigners because they might have the virus”), traumatic stress symptoms (e.g., “I had trouble sleeping because I worried about the virus”), and compulsive checking (e.g., “Seeking reassurance from friends or family about COVID-19”), with each dimension having 6 items. By summing the responses on all items, a total score is used as the measure of CSS and higher scores represent more COVID-19 stress syndrome. Previous studies has checked and established the psychometric properties of the Chinese version of CSS (Tang et al., 2023; Xia et al., 2022). The Cronbach's α of CSS in our sample was 0.94, indicating excellent internal reliability.

*COVID-19 Worry Scale (CWS)*

The CWS (Ahmed et al., 2022) is a unidimensional questionnaire including 7 items (e.g., “How concerned are you about dying from Coronavirus?”), which are rated on a 5-point Likert scale ranging from 1 (never) to 5 (always). It yields a total score and higher scores represent higher worry about COVID-19 infections. The Chinese version of CWS was developed by using a standardized translation and back-translation procedure (Beaton et al., 2000). The Cronbach's α of CWS in our sample was 0.93, indicating excellent internal reliability.

*Death Anxiety Scale (DAS)*

Developed by Templer (1970) to assess anxiety symptoms linked with death, the DAS consists of 15 items that are loaded on a single latent factor. Each item (e.g., “I fear dying a painful death”) is rated on 5-point Likert scale ranging from 1 (strongly disagree) to 5 (strongly agree). A total score can be yielded from all items and higher scores indicate increased death-related anxiety symptoms. The Chinese version of DAS has been confirmed and used in different populations and has shown adequate reliability and validity (Tang et al., 2002; Zhang et al., 2019). The Cronbach's α of DAS in our sample was 0.73, indicating adequate internal reliability.

*Fear of COVID-19 Scale (FCVS)*

The FCVS (Ahorsu et al., 2020) is a unidimensional self-report measure including 7 items, which can yield a total score and higher scores indicate greater fear of COVID-19. Respondents indicate their level of agreement with each item (e.g., “I am afraid of losing my life because of coronavirus-19”) on 5-point Likert scale ranging from 1 (strongly disagree) to 5 (strongly agree). The FCVS has been translated to Chinese version and widely applied in different populations with satisfied psychometric properties (Chen et al., 2022; Chi et al., 2021; Choi et al., 2022). The Cronbach's α of FCVS in our sample was 0.83, indicating satisfactory internal reliability.

*Intolerance of Uncertainty Scale (IUS)*

The IUS (Carleton et al., 2007) is a 12-item self-report questionnaire intended to measure an individual’s predisposition to react to uncertain events or situations. Respondents are asked to rate on each item using a 5-point Likert scale ranging from 1 (not at all characteristic of me) to 5 (entirely characteristic of me). It yields a total score across all items with higher scores representing higher levels of intolerance of uncertainty, although there are two factor structures underlying IUS, i.e., prospective anxiety (e.g., “Unforeseen events upset me greatly”) and inhibitory anxiety (e.g., “Uncertainty keeps me from living a full life”). The stable psychometric properties of the Chinese version of IUS have been successfully demonstrated in different populations (Yang, 2013; Yao et al., 2021; Zhuo et al., 2021). The Cronbach's α of IUS in our sample was 0.89, indicating satisfactory internal reliability.

*Pandemic Grief Scale (PGS)*

The PGS (Lee & Neimeyer, 2022) is a unidimensional 5-item scale designed as a screener to detect possible individuals of dysfunctional grief relating to COVID-19 deaths. Items (e.g., “I wished to die in order to be with the deceased”) are scored on 5-point Likert scale ranging from 1 (not at all) to 5 (nearly every day). The PGS is summed as a total scale score and higher scores represent higher levels of COVID-reduced grief. The Chinese version of PGS was developed by using a standardized translation and back-translation procedure (Beaton et al., 2000). The Cronbach's α of PGS in our sample was 0.80, indicating satisfactory internal reliability.

*PTSD Checklist For DSM-5 (PCL)*

The PCL (Blevins et al., 2015) is a 20-item self-reported measure of posttraumatic stress symptoms based closely on the DSM-5 criteria, including 4 dimensions (i.e., intrusion, avoidance, cognition/mood, and arousal). Respondents rate each item from 1 (not at all) to 5 (extremely) to indicate the degree to which they have been bothered by that particular symptom. The PCL yields a total score and higher scores indicate more posttraumatic stress symptoms. The Chinese version of PCL has been well-validated and widely used to assess COVID-19-related posttraumatic stress symptoms among different populations (Cheng et al., 2020; Liu et al., 2020; Tu et al., 2021). The Cronbach's α of PCL in our sample was 0.94, indicating excellent internal reliability.

*Revised Version of the Impact of Event Scale (IES)*

The IES (Creamer et al., 2003) is a popular instrument for assessing subjective distress caused by traumatic events. It has 22 items and three dimensions (i.e., intrusion, avoidance, and hyperarousal). Respondents are asked to identify a specific stressful life event and indicate how much they were distressed or bothered by each difficulty listed. Items are rated on a 5-point Likert scale ranging from 1 (not at all) to 5 (extremely), with a higher score indicating more severe distress symptoms. The Chinese version of IES has been established and used for investigating pandemic-specific posttraumatic stress symptoms (Peng et al., 2020; Wang et al., 2020). The Cronbach's α of IES in our sample was 0.90, indicating excellent internal reliability.

*Short Version of Health Anxiety Inventory (SHAI)*

Developed by Salkovskis et al. (2002) to assess health-related anxiety symptoms, the SHAI is an 18-item self-rated screening instrument with two sections (i.e., illness likelihood [14 items] and negative consequences [4 items]). Each item includes four statements and participants are asked to select the one which best describes their feelings ranging from 1 (e.g., “I never think I have a serious illness”) to 4 (e.g., “I usually think that I am seriously ill”). The SHAI yields a total score across all items and higher scores represent greater health anxiety. The reliability and validity of the Chinese version of SHAI have been well-established among Chinese populations (Chen et al., 2019; Zhang et al., 2015). The Cronbach's α of SHAI in our sample was 0.86, indicating satisfactory internal reliability.

*Vicarious Traumatization Questionnaire (VTQ)*

The VTQ is a 38-item scale originally developed for investigating Chinese trauma helpers in the 2008 Sichuan earthquake (Han, 2009). It has two facets: physiological responses and psychological responses, and the latter including cognitive responses, behavioral responses, emotional responses and life belief. Individuals are asked to rate how often they felt a certain way after a traumatic even on a 5-point Likert scale from 1 (never) to 5 (always). The total VTQ score can be calculated by summing the responses for all items, with a higher score indicating worse vicarious traumatization. The VTQ has adequate reliability and validity and has been used to assess COVID-related vicarious traumatization in professional and general public populations (Han, 2009; Li et al., 2011; Li et al., 2020). The Cronbach's α of VTQ in our sample was 0.95, indicating excellent internal reliability.

**Reference**

Ahmed, O., Ahmed, M. Z., Alim, S. M. A. H. M., Khan, M. A. U., & Jobe, M. C. (2022). COVID-19 outbreak in Bangladesh and associated psychological problems: An online survey. Death Studies, 46(5), 1080-1089.

Ahorsu, D. K., Lin, C. Y., Imani, V., Saffari, M., Griffiths, M. D., & Pakpour, A. H. (2020). The fear of COVID-19 scale: development and initial validation. International journal of mental health and addiction, 1-9.

Arpaci, I., Karataş, K., & Baloğlu, M. (2020). The development and initial tests for the psychometric properties of the COVID-19 Phobia Scale (C19P-S). Personality and individual differences, 164, 110108.

Beaton, D. E., Bombardier, C., Guillemin, F., & Ferraz, M. B. (2000). Guidelines for the process of cross-cultural adaptation of self-report measures. Spine, 25(24), 3186-3191.

Blevins, C. A., Weathers, F. W., Davis, M. T., Witte, T. K., & Domino, J. L. (2015). The posttraumatic stress disorder checklist for DSM‐5 (PCL‐5): Development and initial psychometric evaluation. Journal of traumatic stress, 28(6), 489-498.

Cai, W., Dong, W., Pan, Y., Wei, C., Zhang, S., Tian, B., ... & Deng, G. (2018). Reliability, validation and norms of the Chinese version of Anxiety Sensitivity Index 3 in a sample of military personnel. PloS one, 13(8), e0201778.

Carleton, R. N., Norton, M. P. J., & Asmundson, G. J. (2007). Fearing the unknown: A short version of the Intolerance of Uncertainty Scale. Journal of anxiety disorders, 21(1), 105-117.

Chen, I. H., Chen, C. Y., Zhao, K. Y., Gamble, J. H., Lin, C. Y., Griffiths, M. D., & Pakpour, A. H. (2022). Psychometric evaluation of fear of COVID-19 Scale (FCV-19S) among Chinese primary and middle schoolteachers, and their students. Current Psychology, 1-17.

Chen, J. H., Tong, K. K., Su, X., Yu, E. W. Y., & Wu, A. M. (2021). Measuring COVID-19 related anxiety and obsession: Validation of the Coronavirus Anxiety Scale and the Obsession with COVID-19 Scale in a probability Chinese sample. Journal of Affective Disorders, 295, 1131-1137.

Chen, Q., Zhang, Y., Zhuang, D., Mao, X., Mi, G., Wang, D., ... & Yuan, Y. (2019). Health anxiety in medical employees: A multicentre study. Journal of International Medical Research, 47(10), 4854-4861.

Cheng, P., Xu, L. Z., Zheng, W. H., Ng, R. M., Zhang, L., Li, L. J., & Li, W. H. (2020). Psychometric property study of the posttraumatic stress disorder checklist for DSM-5 (PCL-5) in Chinese healthcare workers during the outbreak of corona virus disease 2019. Journal of Affective Disorders, 277, 368-374.

Chi, X., Chen, S., Chen, Y., Chen, D., Yu, Q., Guo, T., ... & Zou, L. (2021). Psychometric evaluation of the fear of COVID-19 scale among Chinese population. International Journal of Mental Health and Addiction, 1-16.

Choi, E. P., Duan, W., Fong, D. Y., Lok, K. Y., Ho, M., Wong, J. Y., & Lin, C. C. (2022). Psychometric evaluation of a fear of COVID-19 scale in China: Cross-sectional study. JMIR Formative Research, 6(3), e31992.

Chu, K., Zheng, Y., & Zhu, F. (2022). Psychometric properties of the Chinese version of the COVID-19 Phobia Scale among Chinese undergraduates. Social Behavior and Personality: an international journal, 50(8), 1-7.

Creamer, M., Bell, R., & Failla, S. (2003). Psychometric properties of the impact of event scale—revised. Behaviour research and therapy, 41(12), 1489-1496.

Han, X. (2009). The Construction of Vicarious Traumatization Questionnaire For Trauma Helpers and Its Primary Application. Harbin Engineering University, Harbin, China.

Han, Y., Zhu, J., Li, L., Zhou, H., Li, S., Zhang, J., ... & Zhu, X. (2020). Psychometric properties of the Chinese version of anxiety sensitivity index-3 in women diagnosed with breast cancer. Frontiers in Psychology, 11, 12.

Jiménez, M. P., Rieker, J. A., Reales, J. M., & Ballesteros, S. (2021). COVID-19 peritraumatic distress as a function of age and gender in a Spanish sample. International Journal of Environmental Research and Public Health, 18(10), 5253.

Lee, S. A. (2020). Coronavirus Anxiety Scale: A brief mental health screener for COVID-19 related anxiety. Death studies, 44(7), 393-401.

Lee, S. A., Jobe, M. C., Mathis, A. A., & Gibbons, J. A. (2020). Incremental validity of coronaphobia: Coronavirus anxiety explains depression, generalized anxiety, and death anxiety. Journal of anxiety disorders, 74, 102268.

Lee, S. A., & Neimeyer, R. A. (2022). Pandemic Grief Scale: A screening tool for dysfunctional grief due to a COVID-19 loss. Death Studies, 46(1), 14-24.

Li, L., Yang, Y., Zhang, W. (2011). Relationship among vicarious traumatization, coping styles and social support of trauma helpers. China J. Health Psychol., 19, 412–413.

Li, Z., Ge, J., Yang, M., Feng, J., Qiao, M., Jiang, R., … Yang, C. (2020). Vicarious traumatization in the general public, members, and non-members of medical teams aiding in COVID-19 control. Brain, Behavior, and Immunity, 88, 916–919. doi:10.1016/J.BBI.2020.03.007

Liu, N., Zhang, F., Wei, C., Jia, Y., Shang, Z., Sun, L., ... & Liu, W. (2020). Prevalence and predictors of PTSS during COVID-19 outbreak in China hardest-hit areas: Gender differences matter. Psychiatry research, 287, 112921.

Malach-Pines, A. (2005). The burnout measure, short version. International Journal of Stress Management, 12(1), 78-88.

Marzo, R. R., Singh, A., & Mukti, R. F. (2021). A survey of psychological distress among Bangladeshi people during the COVID-19 pandemic. Clinical epidemiology and global health, 10, 100693.

Nikčević, A. V., & Spada, M. M. (2020). The COVID-19 anxiety syndrome scale: Development and psychometric properties. Psychiatry research, 292, 113322.

Peng, M., Mo, B., Liu, Y., Xu, M., Song, X., Liu, L., ... & Zhang, X. (2020). Prevalence, risk factors and clinical correlates of depression in quarantined population during the COVID-19 outbreak. Journal of affective disorders, 275, 119-124.

Qiu, J., Shen, B., Zhao, M., Wang, Z., Xie, B., & Xu, Y. (2020). A nationwide survey of psychological distress among Chinese people in the COVID-19 epidemic: implications and policy recommendations. General psychiatry, 33(2), e100213.

Salkovskis, P. M., Rimes, K. A., Warwick, H. M. C., & Clark, D. (2002). The Health Anxiety Inventory: development and validation of scales for the measurement of health anxiety and hypochondriasis. Psychological medicine, 32(5), 843-853.

Shrestha, D. B., Thapa, B. B., Katuwal, N., Shrestha, B., Pant, C., Basnet, B., ... & Rouniyar, R. (2020). Psychological distress in Nepalese residents during COVID-19 pandemic: a community level survey. BMC psychiatry, 20(1), 491.

Tang, C. S. K., Wu, A. M., & W. Yan, E. C. (2002). Psychosocial correlates of death anxiety among Chinese college students. Death studies, 26(6), 491-499.

Tang, Y. M., Wu, T. L., & Liu, H. T. (2023). Causal Model Analysis of the Effect of Formalism, Fear of Infection, COVID-19 Stress on Firefighters’ Post-Traumatic Stress Syndrome and Insomnia. International Journal of Environmental Research and Public Health, 20(2), 1097.

Taylor, S., Landry, C. A., Paluszek, M. M., Fergus, T. A., McKay, D., & Asmundson, G. J. (2020). Development and initial validation of the COVID Stress Scales. Journal of anxiety disorders, 72, 102232.

Taylor, S., Zvolensky, M. J., Cox, B. J., Deacon, B., Heimberg, R. G., Ledley, D. R., ... & Cardenas, S. J. (2007). Robust dimensions of anxiety sensitivity: development and initial validation of the Anxiety Sensitivity Index-3. Psychological assessment, 19(2), 176-188.

Templer, D. I. (1970). The construction and validation of a death anxiety scale. The Journal of general psychology, 82(2), 165-177.

Tu, Y., Zhang, Y., Li, Y., Zhao, Q., Bi, Y., Lu, X., ... & Hu, L. (2021). Post-traumatic stress symptoms in COVID-19 survivors: a self-report and brain imaging follow-up study. Molecular Psychiatry, 26(12), 7475-7480.

Wang, C., Pan, R., Wan, X., Tan, Y., Xu, L., McIntyre, R. S., ... & Ho, C. (2020). A longitudinal study on the mental health of general population during the COVID-19 epidemic in China. Brain, behavior, and immunity, 87, 40-48.

Wang, L., Liu, W. T., Zhu, X. Z., Wang, Y. P., Li, L. Y., Yang, Y. L., & Ryder, A. G. (2014). Validity and reliability of the Chinese Version of the Anxiety Sensitivity Index-3 in healthy adult women. Chinese Mental Health Journal, 28(10), 767–771.

Wang, R., Ye, B., Wang, P., Tang, C., & Yang, Q. (2022). Coronavirus stress and overeating: the role of anxiety and COVID-19 burnout. Journal of Eating Disorders, 10(1), 1-8.

Xia, L., Lian, Q., Yang, H., & Wu, D. (2022). The adaption of the Chinese version of the COVID Stress Scales as a screening instrument of stress: Psychometric properties during the COVID-19 pandemic. Frontiers in Public Health, 10, 962304.

Xin, L., Wang, L., Cao, X., Tian, Y., Yang, Y., Wang, K., ... & Wu, Q. (2022). Prevalence and influencing factors of pandemic fatigue among Chinese public in Xi'an city during COVID-19 new normal: a cross-sectional study. Frontiers in Public Health, 10, 971115-971115.

Yang, Z. (2013). Psychometric properties of the Intolerance of Uncertainty Scale (IUS) in a Chinese-speaking population. Behavioural and Cognitive Psychotherapy, 41(4), 500-504.

Yao, N., Qian, M., Jiang, Y., & Elhai, J. D. (2021). The influence of intolerance of uncertainty on anxiety and depression symptoms in Chinese-speaking samples: Structure and validity of the Chinese translation of the Intolerance of Uncertainty Scale. Journal of personality assessment, 103(3), 406-415.

Ye, B., Chen, X., Zhang, Y., & Yang, Q. (2022). Psychological flexibility and COVID-19 burnout in Chinese college students: A moderated mediation model. Journal of Contextual Behavioral Science, 24, 126-133.

Yıldırım, M., & Solmaz, F. (2022). COVID-19 burnout, COVID-19 stress and resilience: Initial psychometric properties of COVID-19 Burnout Scale. Death Studies, 46(3), 524-532.

Zhang, J., Peng, J., Gao, P., Huang, H., Cao, Y., Zheng, L., & Miao, D. (2019). Relationship between meaning in life and death anxiety in the elderly: self-esteem as a mediator. BMC geriatrics, 19, 1-8.

Zhang, Y., Liu, R., Li, G., Mao, S., & Yuan, Y. (2015). The reliability and validity of a Chinese-version Short Health Anxiety Inventory: an investigation of university students. Neuropsychiatric Disease and Treatment, 1739-1747.

Zhao, B., Xu, J., Kong, F., & Nam, E. W. (2023). Validation of the chinese version of the COVID-19 phobia scale among chinese college students. Heliyon, doi: https://doi.org/10.1016/j.heliyon.2023.e13468.

Zhuo, L., Wu, Q., Le, H., Li, H., Zheng, L., Ma, G., & Tao, H. (2021). COVID-19-related intolerance of uncertainty and mental health among back-to-school students in Wuhan: the moderation effect of social support. International journal of environmental research and public health, 18(3), 981.

**Supplementary Tables and Figures**

**
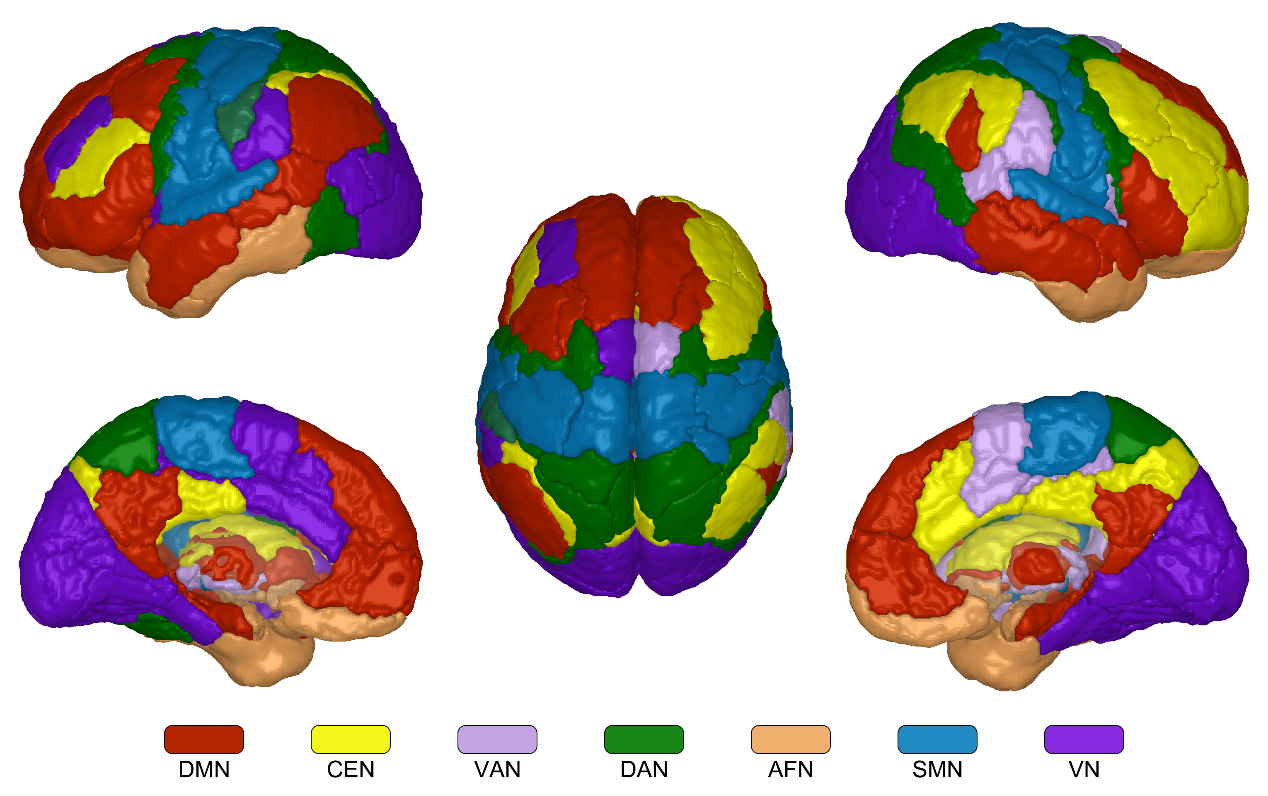
**

**Figure S1.** Brain functional network parcellation. The cortical and subcortical brain areas were parceled with 136 seed regions, and these seed regions were assigned to 7 macroscale networks in accordance with priori hypothesis: the default mode network (DMN), central executive network (CEN), ventral attention network (VAN), dorsal attention network (DAN), cortical affective network (AFN), sensorimotor network (SMN) and visual network (VN). Brain atlas is available online at https://osf.io/2k4ex/, and the corresponding information of each seed regions is shown in Table S2.


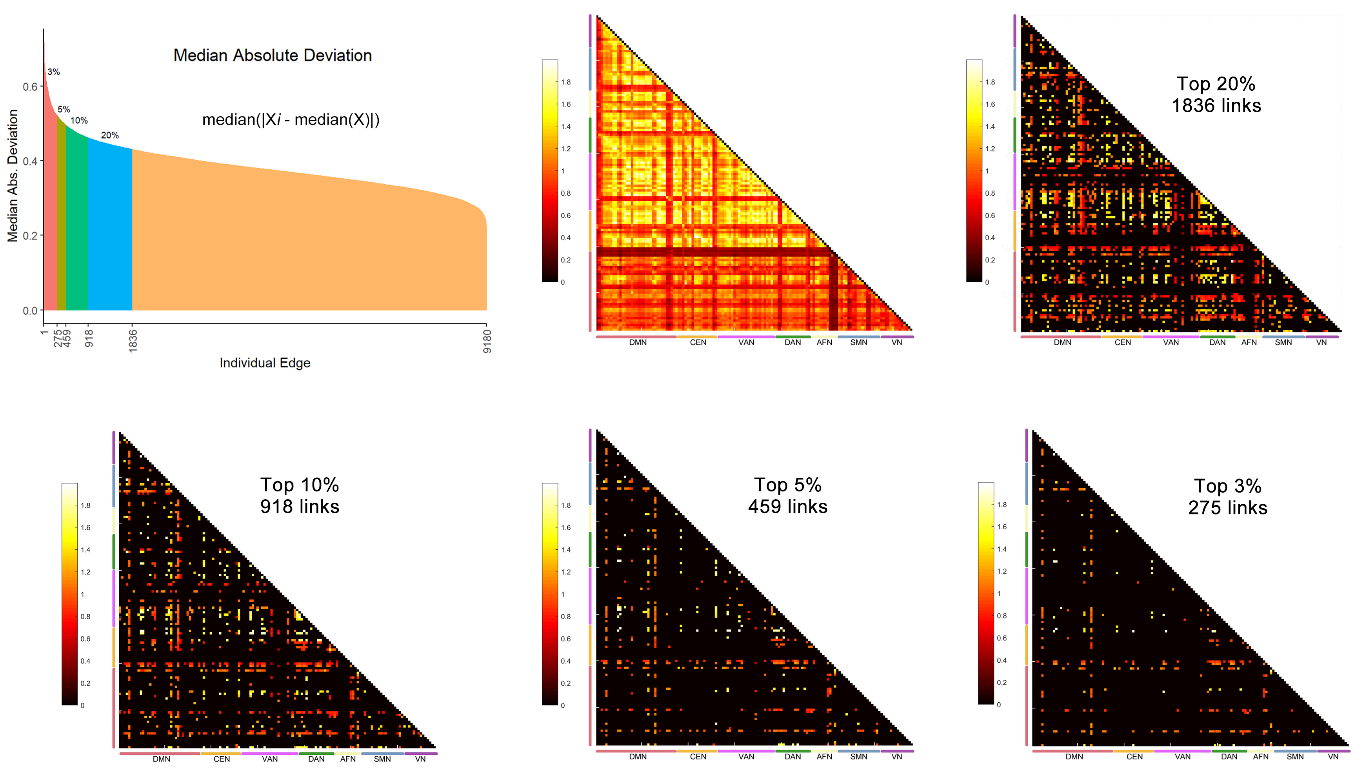


**Figure S2.** Feature Selection Based on Median Absolute Deviation (MAD). Given that regularized CCA tends to capture sources of variation derived from both datasets, we selected the top 5% or 459 connectivity links that were variable computed by MAD in our sample. MAD of each link strength is presented in the first panel in decreasing order, and the top 3%, 5%, 10% and 20% are labeled. The average connectivity matrix across all subjects of links with MAD at the top 3%, 5%, 10% and 20% levels are shown in the other panels.


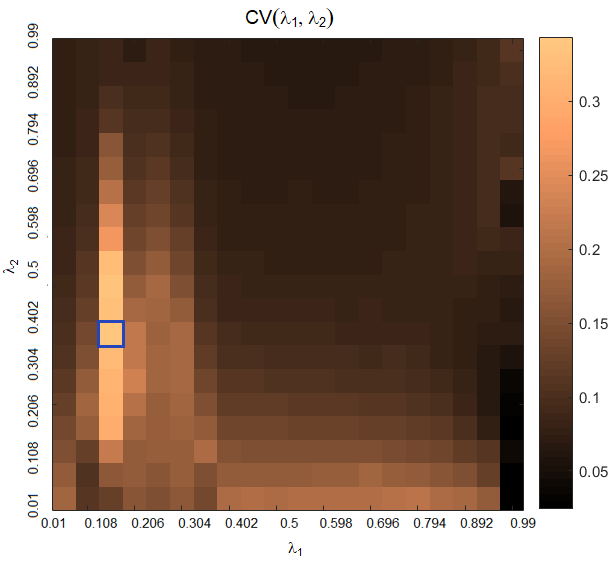


**Figure S3.** Grid Search for Regularization Parameters. We evaluated and optimized various pairs of regularization parameters by grid search in a constrained range between 0.01 and 0.99 (length = 20) to yield the highest canonical correlation of the first variate.


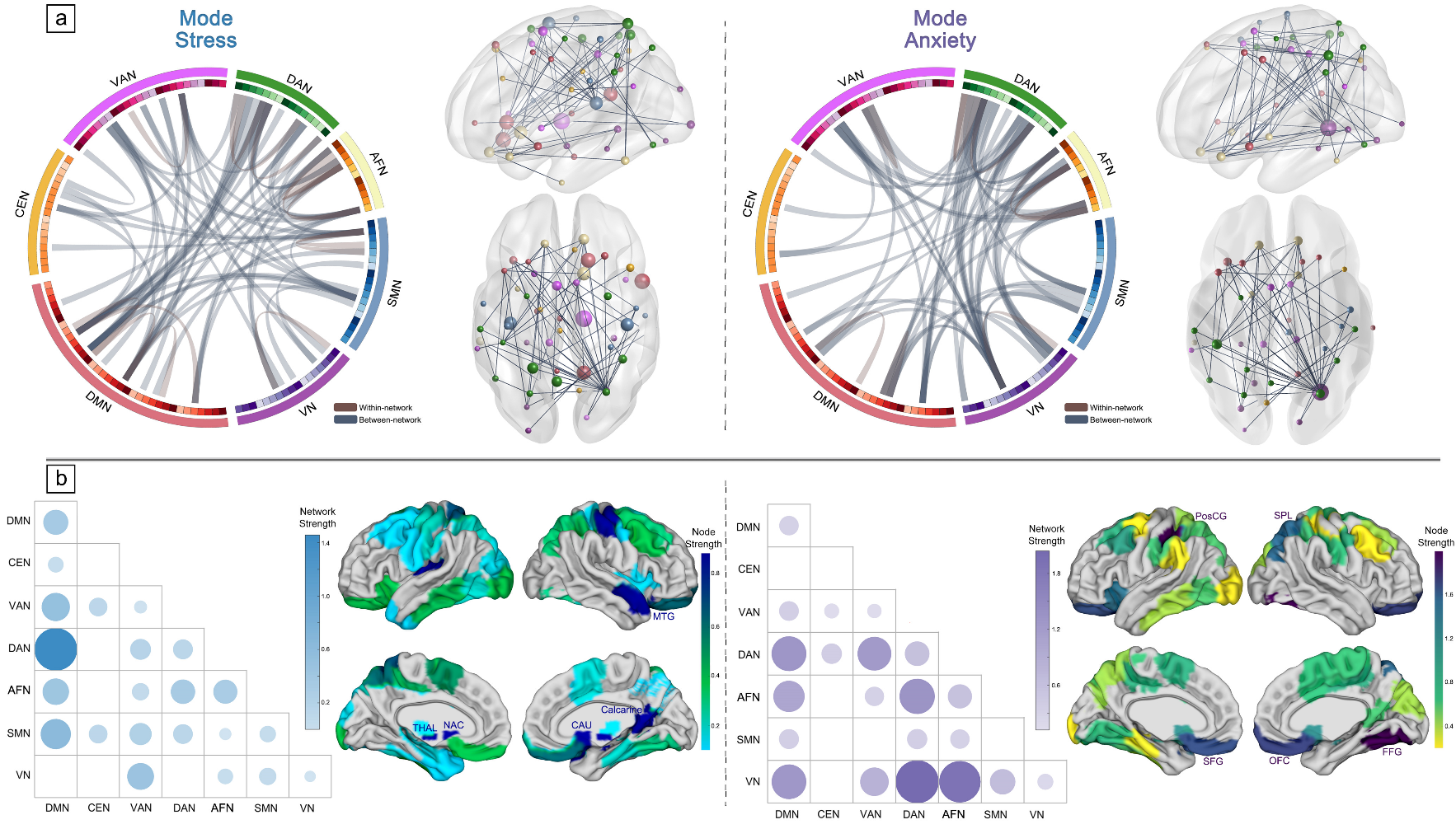


**Figure S4.** Mode-specific Brain Connectivity Patterns. (a) Identified neural connectivity patterns with mode-specific edges that stably contributed to each mode, delineated by Circos plots and ball-and-stick plots of the brain. (b) Identified mode-specific brain connectome patterns at the network community level and nodal level with the neuroanatomical distribution.


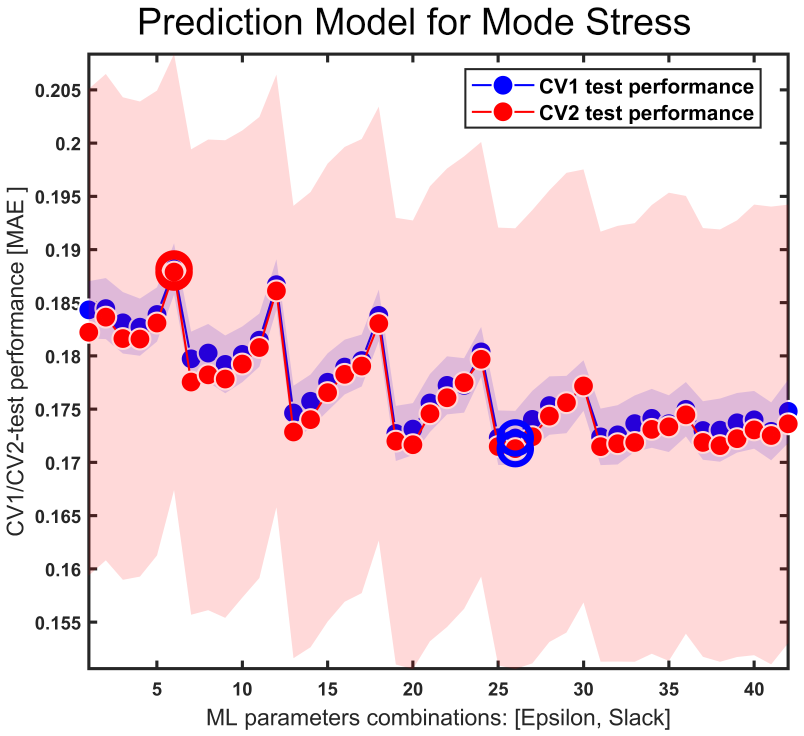

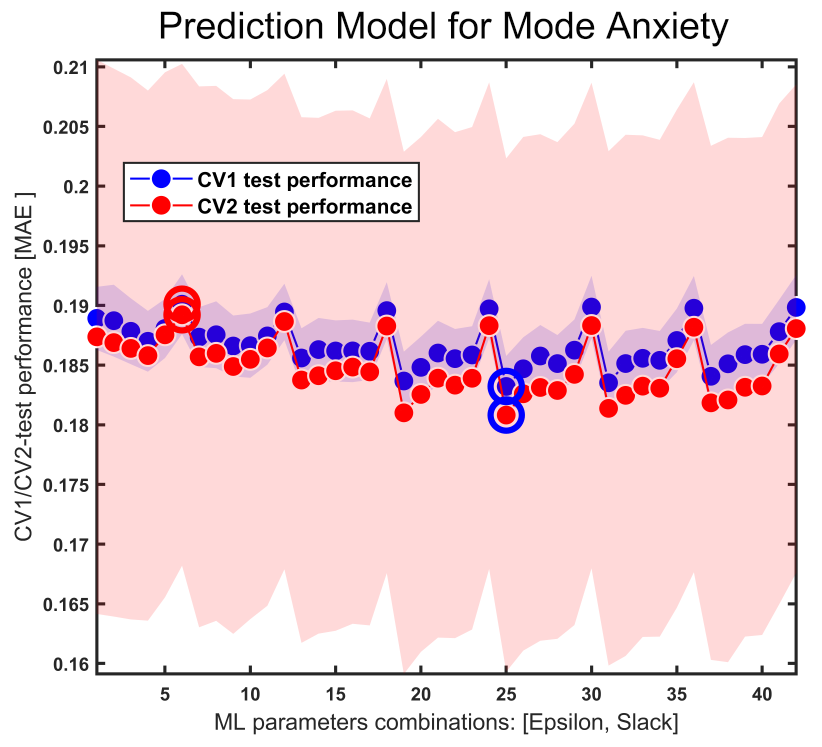


**Figure S5.** Parameter Search for Nested Cross-validation. We used a linear support vector machine (SVM) that underwent greedy forward search for optimized parameters (Epsilon and Slack) to create fitting models that optimally predict the observed scores.


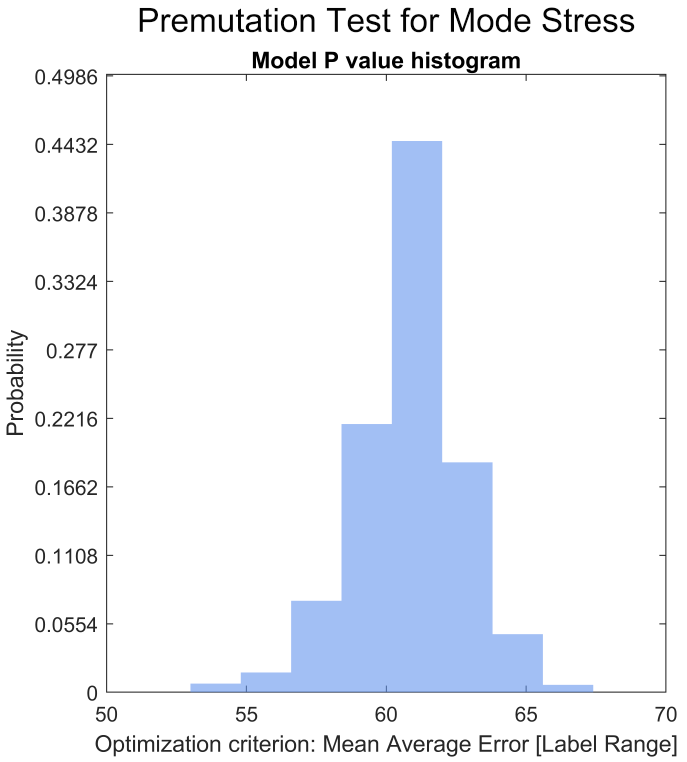

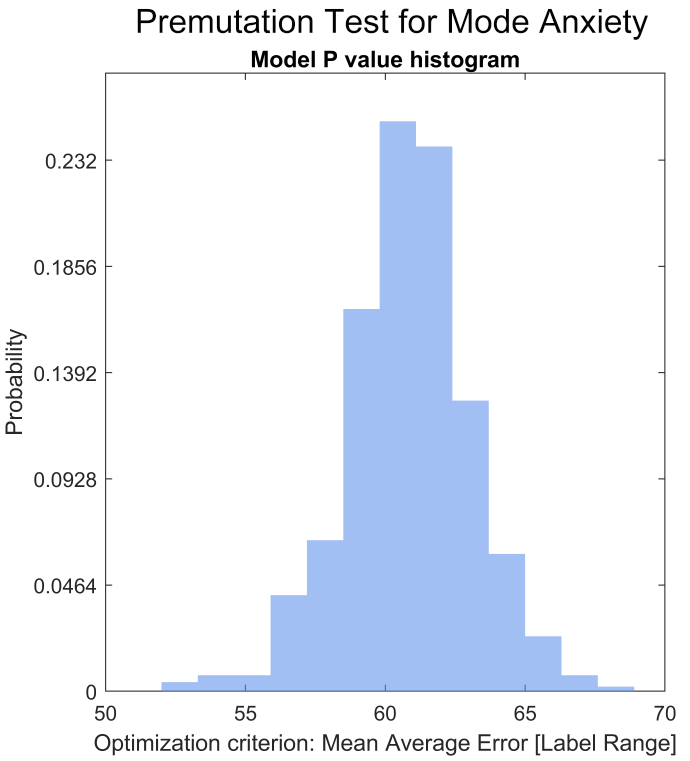


**Figure S6.** Permutation Test for Prediction Models. Histograms of mean average error (MAE) for trained classifier are exhibited. Permutation tests (1000 times on label) were applied to inform whether our predictive model was significantly different from those with respective ensemble predictors (*p* < .01), and we evaluated our models by calculating MAE. The MAE of the predictive model for mode stress is 5.1 and that for mode anxiety is 5.4, and we could conclude that our predictive models are significant rather by chance.


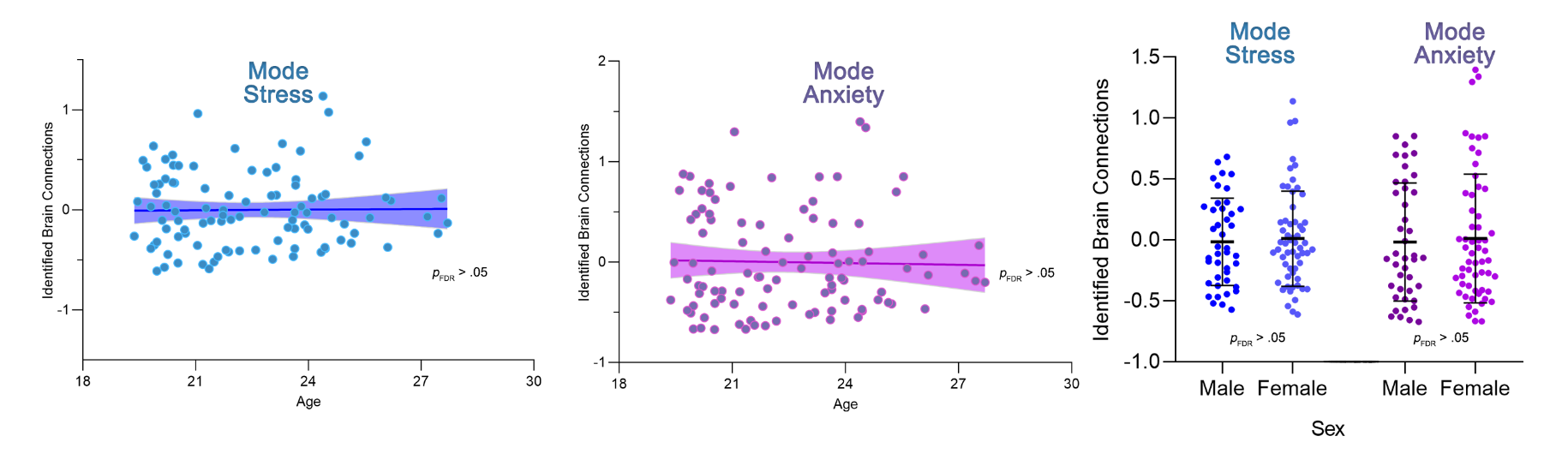


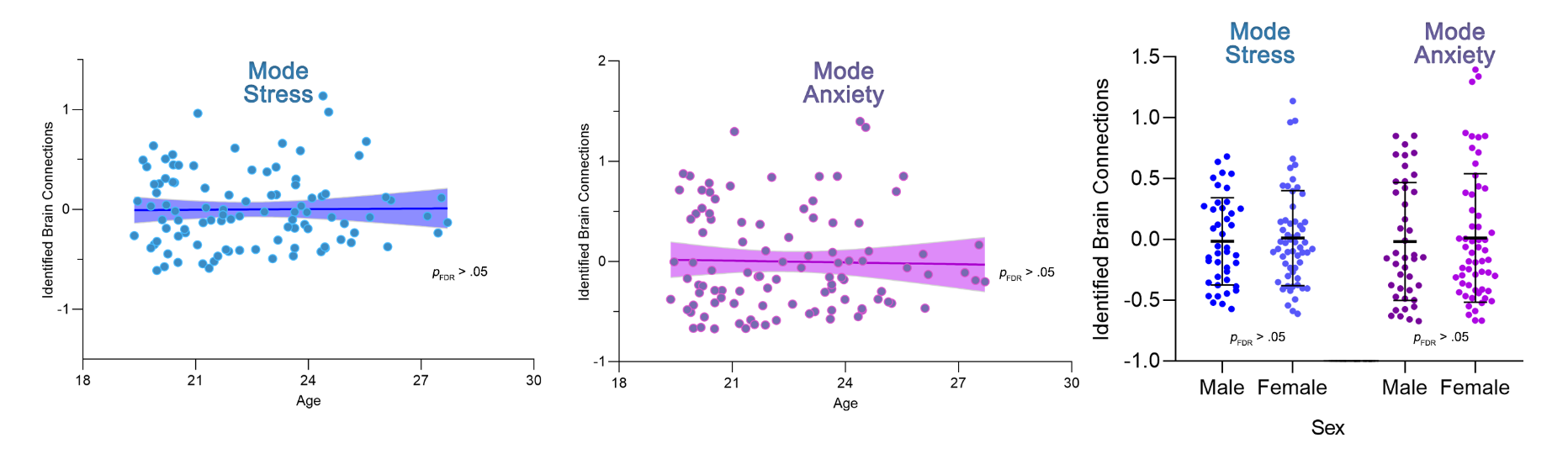


**Figure S7.** Developmental Effects and Sex Differences. Brain connectivity patterns of mode stress and mode anxiety are not significantly correlated with age, and no sex differences are identified in both modes.

**Table S1.** Seed Regions of Brain Functional Parcellation.

| Label | Peak Coordinate (MNI) | | | Anatomical Description | Network |
| --- | --- | --- | --- | --- | --- |
|  | X | Y | Z |  |  |
| 1 | -26 | -34 | -17 | Fusiform_L | VN |
| 2 | -26 | -77 | -14 | Fusiform_L | VN |
| 3 | -17 | -60 | -7 | Lingual_L | VN |
| 4 | -27 | -95 | -4 | Occipital_Inf_L | VN |
| 5 | -5 | -92 | -2 | Calcarine_L | VN |
| 6 | -12 | -67 | 7 | Calcarine_L | VN |
| 7 | -47 | -71 | 11 | Occipital_Mid_L | VN |
| 8 | -25 | -88 | 20 | Occipital_Mid_L | VN |
| 9 | -6 | -82 | 26 | Cuneus_L | VN |
| 10 | -53 | -23 | 8 | Temp_Sup_L | SMN |
| 11 | -37 | -21 | 16 | Insula_L | SMN |
| 12 | -54 | -12 | 13 | Temp_Sup_L | SMN |
| 13 | -55 | -8 | 34 | Postcentral_L | SMN |
| 14 | -39 | -23 | 59 | Precentral_L | SMN |
| 15 | -6 | -29 | 70 | Paracentral_Lobule_L | SMN |
| 16 | -47 | -58 | -13 | Occipital_Inf_L | DAN |
| 17 | -57 | -25 | 39 | SupraMarginal_L | DAN |
| 18 | -24 | -68 | 49 | Par_Sup_L | DAN |
| 19 | -42 | -34 | 48 | Postcentral_L | DAN |
| 20 | -6 | -60 | 56 | Precuneus_L | DAN |
| 21 | -22 | -51 | 66 | Par_Sup_L | DAN |
| 22 | -48 | 6 | 28 | Frontal_Inf_Oper_L | DAN |
| 23 | -26 | -3 | 59 | Frontal_Mid_L | DAN |
| 24 | -59 | -38 | 29 | SupraMarginal_L | VAN |
| 25 | -41 | -1 | -7 | Insula_L | VAN |
| 26 | -38 | 12 | 6 | Insula_L | VAN |
| 27 | -30 | 44 | 30 | Frontal_Mid_L | VAN |
| 28 | -5 | 20 | 34 | Cingulum_Mid_L | VAN |
| 29 | -11 | -34 | 45 | Cingulum_Mid_L | VAN |
| 30 | -6 | 4 | 62 | Supp_Motor_Area_L | VAN |
| 31 | -14 | 32 | -20 | Frontal_Sup_Orb_L | LN |
| 32 | -32 | 2 | -37 | Temp_Inf_L | LN |
| 33 | -57 | -33 | -21 | Temp_Inf_L | LN |
| 34 | -37 | -53 | 46 | Par_Inf_L | CEN |
| 35 | -43 | 33 | 21 | Frontal_Inf_Tri_L | CEN |
| 36 | -9 | -73 | 38 | Precuneus_L | CEN |
| 37 | -4 | -26 | 33 | Cingulum_Mid_L | CEN |
| 38 | -55 | -4 | -20 | Temp_Mid_L | DMN |
| 39 | -58 | -32 | -1 | Temp_Mid_L | DMN |
| 40 | -57 | -50 | 12 | Temp_Mid_L | DMN |
| 41 | -48 | -63 | 35 | Angular_L | DMN |
| 42 | -35 | 21 | -11 | Insula_L | DMN |
| 43 | -47 | 33 | -3 | Frontal_Inf_Orb_L | DMN |
| 44 | -6 | 47 | 0 | Cingulum_Ant_L | DMN |
| 45 | -24 | 61 | -1 | Frontal_Sup_Orb_L | DMN |
| 46 | -9 | 48 | 41 | Frontal_Sup_Medial_L | DMN |
| 47 | -41 | 14 | 48 | Frontal_Mid_L | DMN |
| 48 | -25 | 20 | 51 | Frontal_Mid_L | DMN |
| 49 | -11 | -56 | 13 | Precuneus_L | DMN |
| 50 | -6 | -53 | 33 | Cingulum_Post_L | DMN |
| 51 | 32 | -31 | -22 | Fusiform_R | VN |
| 52 | 27 | -66 | -12 | Fusiform_R | VN |
| 53 | 49 | -60 | -11 | Temp_Inf_R | VN |
| 54 | 22 | -96 | -5 | Calcarine_R | VN |
| 55 | 8 | -76 | 5 | Calcarine_R | VN |
| 56 | 17 | -57 | 5 | Calcarine_R | VN |
| 57 | 36 | -82 | 16 | Occipital_Mid_R | VN |
| 58 | 13 | -86 | 29 | Cuneus_R | VN |
| 59 | 53 | -16 | 7 | Temp_Sup_R | SMN |
| 60 | 40 | -15 | 15 | Insula_R | SMN |
| 61 | 57 | -4 | 11 | Rolandic_Oper_R | SMN |
| 62 | 58 | -5 | 31 | Postcentral_R | SMN |
| 63 | 47 | -11 | 48 | Precentral_R | SMN |
| 64 | 41 | -22 | 60 | Precentral_R | SMN |
| 65 | 30 | -37 | 64 | Postcentral_R | SMN |
| 66 | 6 | -26 | 70 | Paracentral_Lobule_R | SMN |
| 67 | 50 | -62 | 16 | Temp_Mid_R | DAN |
| 68 | 50 | -24 | 42 | Postcentral_R | DAN |
| 69 | 38 | -45 | 49 | Par_Inf_R | DAN |
| 70 | 27 | -67 | 51 | Par_Sup_R | DAN |
| 71 | 14 | -52 | 66 | Par_Sup_R | DAN |
| 72 | 49 | 10 | 27 | Frontal_Inf_Oper_R | DAN |
| 73 | 28 | -3 | 59 | Frontal_Sup_R | DAN |
| 74 | 58 | -42 | 13 | Temp_Sup_R | VAN |
| 75 | 61 | -26 | 27 | SupraMarginal_R | VAN |
| 76 | 40 | 8 | 1 | Insula_R | VAN |
| 77 | 11 | -31 | 45 | Cingulum_Mid_R | VAN |
| 78 | 7 | 6 | 52 | Supp_Motor_Area_R | VAN |
| 79 | 12 | 35 | -20 | Rectus_R | LN |
| 80 | 38 | 1 | -38 | Temp_Inf_R | LN |
| 81 | 57 | -39 | 44 | SupraMarginal_R | CEN |
| 82 | 45 | -63 | 46 | Angular_R | CEN |
| 83 | 30 | 58 | -3 | Frontal_Sup_Orb_R | CEN |
| 84 | 45 | 39 | 15 | Frontal_Inf_Tri_R | CEN |
| 85 | 32 | 46 | 29 | Frontal_Mid_R | CEN |
| 86 | 43 | 16 | 45 | Frontal_Mid_R | CEN |
| 87 | 5 | -27 | 33 | Cingulum_Mid_R | CEN |
| 88 | 6 | 28 | 30 | Cingulum_Ant_R | CEN |
| 89 | 9 | -66 | 43 | Precuneus_R | CEN |
| 90 | 55 | -51 | 31 | Angular_R | DMN |
| 91 | 62 | -23 | -19 | Temp_Inf_R | DMN |
| 92 | 51 | 7 | -18 | Temp_Pole_Mid_R | DMN |
| 93 | 57 | -26 | -2 | Temp_Mid_R | DMN |
| 94 | 35 | 26 | -15 | Frontal_Inf_Orb_R | DMN |
| 95 | 51 | 28 | 0 | Frontal_Inf_Tri_R | DMN |
| 96 | 7 | 48 | 1 | Frontal_Sup_Medial_R | DMN |
| 97 | 11 | 50 | 39 | Frontal_Sup_Medial_R | DMN |
| 98 | 26 | 24 | 50 | Frontal_Sup_R | DMN |
| 99 | 12 | -54 | 14 | Calcarine_R | DMN |
| 100 | 7 | -52 | 31 | Cingulum_Post_R | DMN |
| 101 | 21 | 2 | -22 | AMYG_medial_L | LN |
| 102 | 24 | 1 | -20 | AMYG_medial_R | LN |
| 103 | -26 | -2 | -15 | AMYG_lateral_L | LN |
| 104 | 27 | 0 | -25 | AMYG_lateral_R | LN |
| 105 | -12 | 21 | -1 | vCaudate_L | DMN |
| 106 | 15 | 21 | -1 | vCaudate_R | DMN |
| 107 | -26 | -4 | 13 | GlobusPallidus_L | VAN |
| 108 | 27 | -4 | 12 | GlobusPallidus_R | VAN |
| 109 | -19 | -1 | -8 | NucleusAccumbens_L | LN |
| 110 | 12 | 12 | -8 | NucleusAccumbens_R | LN |
| 111 | -21 | 12 | -1 | vmPutaman_L | VAN |
| 112 | 23 | 12 | 1 | vmPutamen_R | VAN |
| 113 | -17 | -12 | 23 | dCaudate_L | CEN |
| 114 | 16 | -5 | 23 | dCaudate_R | CEN |
| 115 | -29 | -13 | 9 | dlPutamen_L | SMN |
| 116 | 27 | 1 | 1 | dlPutamen_R | SMN |
| 117 | -19 | -13 | -22 | HIP_rostral_L | DMN |
| 118 | 25 | -13 | -16 | HIP_rostral_R | DMN |
| 119 | -20 | -40 | 3 | HIP_caudal_L | DMN |
| 120 | 30 | -33 | -8 | HIP_caudal_R | DMN |
| 121 | -4 | -18 | 5 | THAL_mPF_L | DMN |
| 122 | 6 | -18 | 9 | THAL_mPF_R | DMN |
| 123 | -21 | -18 | 6 | THAL_mPM_L | VAN |
| 124 | 12 | -18 | -2 | THAL_mPM_R | VAN |
| 125 | -15 | -24 | -2 | THAL_sensory_L | SMN |
| 126 | 18 | -24 | 2 | THAL_sensory_R | SMN |
| 127 | -2 | -13 | 5 | THAL_rostral_Temp_L | DMN |
| 128 | 0 | -13 | 7 | THAL_rostral_Temp_R | DMN |
| 129 | -14 | -24 | 9 | THAL_posterior_Par_L | VAN |
| 130 | 13 | -18 | 15 | THAL_posterior_Par_R | VAN |
| 131 | -13 | -11 | 15 | THAL_occipital_L | VAN |
| 132 | 11 | -28 | 9 | THAL_occipital_R | VAN |
| 133 | -12 | -22 | 15 | THAL_caudal_Temp_L | CEN |
| 134 | 10 | -10 | 15 | THAL_caudal_Temp_R | CEN |
| 135 | -15 | -19 | 9 | THAL_lateral_PF_L | VAN |
| 136 | 13 | -12 | 9 | THAL_lateral_PF_R | VAN |

Note: The anatomical description for each seed region was derived based on Automated anatomical labelling atlas 3 (Ref: Rolls E T, Huang C C, Lin C P, et al. Automated anatomical labelling atlas 3. Neuroimage, 2020, 206: 116189).

Abbreviation: MNI = Montreal Neurological Institute template, L=left, R = right, Inf = inferior, Mid = middle, Sup = superior, Supp = supplementary, Orb = orbital, Tri = triangular, Ant = anterior, Post = posterior, Oper = opercular, AMYG = amygdala, vm = ventromedial, dl = dorsolateral, HIP = hippocampus, THAL = thalamus, Temp = temporal, Par = parietal, PF = prefrontal, PM = premotor.

**Table S2.** Descriptive Statistics and Bivariate Correlations of Pandemic-specific Psychological Measures

| Measure | Mean ± SD | Range | 1 | 2 | 3 | 4 | 5 | 6 | 7 | 8 | 9 | 10 | 11 | 12 | 13 | 14 | 15 | 16 | 17 |
| --- | --- | --- | --- | --- | --- | --- | --- | --- | --- | --- | --- | --- | --- | --- | --- | --- | --- | --- | --- |
| 1. CPDI | 34.50 ± 9.73 | 24–70 |  | .535^**^ | .329^**^ | .363^**^ | .594^**^ | .455^**^ | .345^**^ | .520^**^ | .539^**^ | .588^**^ | .568^**^ | .685^**^ | .296^**^ | .539^**^ | .355^**^ | .542^**^ | .698^**^ |
| 2. CASS | 13.59 ± 4.46 | 9–24 | .538^**^ |  | .715^**^ | .394^**^ | .605^**^ | .590^**^ | .195 | .546^**^ | .554^**^ | .688^**^ | .695^**^ | .384^**^ | .122 | .199^*^ | .303^**^ | .271^**^ | .514^**^ |
| 3. CRSBS | 7.20 ± 2.73 | 5–16 | .335^**^ | .719^**^ |  | .535^**^ | .510^**^ | .484^**^ | .100 | .313^**^ | .363^**^ | .418^**^ | .470^**^ | .190 | .035 | .129 | .138 | .081 | .292^**^ |
| 4. CAS | 5.64 ± 1.63 | 5–14 | .360^**^ | .391^**^ | .530^**^ |  | .458^**^ | .228^*^ | .161 | .401^**^ | .352^**^ | .217^*^ | .254^*^ | .214^*^ | .015 | .186 | .157 | .171 | .277^**^ |
| 5. CBS | 12.15 ± 3.54 | 10–23 | .593^**^ | .594^**^ | .499^**^ | .453^**^ |  | .557^**^ | .269^**^ | .468^**^ | .629^**^ | .670^**^ | .614^**^ | .446^**^ | .181 | .357^**^ | .364^**^ | .495^**^ | .521^**^ |
| 6. CWS | 11.21 ± 4.44 | 7–21 | .448^**^ | .578^**^ | .471^**^ | .227^*^ | .559^**^ |  | .321^**^ | .285^**^ | .611^**^ | .652^**^ | .726^**^ | .315^**^ | .200^*^ | .308^**^ | .347^**^ | .266^**^ | .391^**^ |
| 7. SHAI | 33.90 ± 6.29 | 23–50 | .354^**^ | .199^*^ | .106 | .158 | .278^**^ | .319^**^ |  | .070 | .355^**^ | .294^**^ | .337^**^ | .247^*^ | .431^**^ | .548^**^ | .494^**^ | .287^**^ | .289^**^ |
| 8. PGS | 6.32 ± 2.13 | 5–15 | .505^**^ | .535^**^ | .302^**^ | .401^**^ | .455^**^ | .284^**^ | .058 |  | .422^**^ | .482^**^ | .490^**^ | .444^**^ | .162 | .257^*^ | .231^*^ | .437^**^ | .626^**^ |
| 9. FCVS | 9.71 ± 3.60 | 7–22 | .522^**^ | .521^**^ | .333^**^ | .343^**^ | .634^**^ | .611^**^ | .354^**^ | .407^**^ |  | .713^**^ | .655^**^ | .419^**^ | .169 | .410^**^ | .460^**^ | .466^**^ | .621^**^ |
| 10. CPS | 31.82 ± 10.31 | 20–57 | .580^**^ | .674^**^ | .404^**^ | .216^*^ | .671^**^ | .655^**^ | .293^**^ | .479^**^ | .712^**^ |  | .854^**^ | .551^**^ | .233^*^ | .430^**^ | .457^**^ | .474^**^ | .594^**^ |
| 11. CSS | 61.52 ± 18.34 | 36–100 | .561^**^ | .687^**^ | .461^**^ | .254^*^ | .610^**^ | .726^**^ | .332^**^ | .490^**^ | .645^**^ | .853^**^ |  | .548^**^ | .352^**^ | .432^**^ | .459^**^ | .443^**^ | .532^**^ |
| 12. VTQ | 67.40 ± 21.46 | 38–112 | .688^**^ | .385^**^ | .194 | .212^*^ | .451^**^ | .313^**^ | .259^**^ | .430^**^ | .415^**^ | .547^**^ | .543^**^ |  | .487^**^ | .545^**^ | .395^**^ | .548^**^ | .557^**^ |
| 13. IUS | 34.98 ± 9.01 | 12–50 | .309^**^ | .130 | .048 | .013 | .192 | .196 | .444^**^ | .144 | .170 | .229^*^ | .343^**^ | .496^**^ |  | .676^**^ | .385^**^ | .226^*^ | .298^**^ |
| 14. ASI | 39.90 ± 13.11 | 18–69 | .546^**^ | .203^*^ | .136 | .181 | .367^**^ | .304^**^ | .559^**^ | .236^*^ | .409^**^ | .425^**^ | .422^**^ | .554^**^ | .687^**^ |  | .459^**^ | .386^**^ | .495^**^ |
| 15. DAS | 43.24 ± 7.83 | 24–66 | .352^**^ | .284^**^ | .122 | .151 | .382^**^ | .352^**^ | .497^**^ | .213^*^ | .487^**^ | .461^**^ | .449^**^ | .399^**^ | .391^**^ | .469^**^ |  | .314^**^ | .412^**^ |
| 16. PCL | 24.88 ± 7.62 | 20–59 | .536^**^ | .265^**^ | .075 | .171 | .495^**^ | .269^**^ | .285^**^ | .435^**^ | .465^**^ | .477^**^ | .444^**^ | .545^**^ | .222^*^ | .380^**^ | .315^**^ |  | .551^**^ |
| 17. IES | 28.94 ± 7.59 | 22–60 | .688^**^ | .500^**^ | .278^**^ | .276^**^ | .524^**^ | .396^**^ | .287^**^ | .621^**^ | .624^**^ | .598^**^ | .532^**^ | .553^**^ | .292^**^ | .488^**^ | .419^**^ | .553^**^ |  |

Note: SD = standard deviation, CPDI = COVID-19 Peritraumatic Distress Index, CASS = COVID-19 Anxiety Syndrome Scale, CRSBS = Coronavirus Reassurance-Seeking Behaviors Scale, CAS = Coronavirus Anxiety Scale, CBS = COVID-19 Burnout Scale, CWS = COVID-19 Worry Scale, SHAI = Short Version of Health Anxiety Inventory, PGS = Pandemic Grief Scale, FCVS = Fear of COVID-19 Scale, CPS = COVID-19 Phobia Scale, CSS = COVID-19 Stress Scales, VTQ = Vicarious Traumatization Questionnaire, IUS = Intolerance of Uncertainty Scale, ASI = Anxiety Sensitivity Index, DAS = Death Anxiety Scale, PCL = PTSD Checklist For DSM-5, IES = Revised Version of the Impact of Event Scale. The valuables above the diagonal line indicate the Pearson's correlation coefficients with sex and age as covariates; the valuables below the diagonal line indicate the Pearson's correlation coefficients with no covariates. ^*^ p < 0.05, ^**^ p < 0.01.

**Table S3.** Identified brain connectome patterns at the nodal level.

| Label | Peak Coordinate (MNI) | | | Anatomical Description | Network | Node Strength in Mode Stress | Node Strength in Mode Anxiety |
| --- | --- | --- | --- | --- | --- | --- | --- |
|  | X | Y | Z |  |  |  |  |
| 1 | -26 | -34 | -17 | Fusiform_L | VN | 0.098 | 0.254 |
| 2 | -26 | -77 | -14 | Fusiform_L | VN | 0.421 | 1.397 |
| 3 | -17 | -60 | -7 | Lingual_L | VN | / | 0.672 |
| 4 | -27 | -95 | -4 | Occipital_Inf_L | VN | 0.37 | 0.223 |
| 8 | -25 | -88 | 20 | Occipital_Mid_L | VN | 0.146 | / |
| 11 | -37 | -21 | 16 | Insula_L | SMN | 0.888 | / |
| 13 | -55 | -8 | 34 | Postcentral_L | SMN | 0.122 | / |
| 14 | -39 | -23 | 59 | Precentral_L | SMN | 0.214 | / |
| 16 | -47 | -58 | -13 | Occipital_Inf_L | DAN | 0.746 | 1.386 |
| 17 | -57 | -25 | 39 | SupraMarginal_L | DAN | 0.202 | 0.616 |
| 18 | -24 | -68 | 49 | Par_Sup_L | DAN | 0.201 | 0.588 |
| 19 | -42 | -34 | 48 | Postcentral_L | DAN | 0.681 | 3.355 |
| 20 | -6 | -60 | 56 | Precuneus_L | DAN | 1.57 | 2.087 |
| 21 | -22 | -51 | 66 | Par_Sup_L | DAN | 0.895 | 0.841 |
| 23 | -26 | -3 | 59 | Frontal_Mid_L | DAN | 0.369 | 0.639 |
| 24 | -59 | -38 | 29 | SupraMarginal_L | VAN | 0.089 | 0.241 |
| 29 | -11 | -34 | 45 | Cingulum_Mid_L | VAN | 0.614 | 1.449 |
| 30 | -6 | 4 | 62 | Supp_Motor_Area_L | VAN | 0.621 | 1.084 |
| 31 | -14 | 32 | -20 | Frontal_Sup_Orb_L | LN | 0.735 | 2.304 |
| 32 | -32 | 2 | -37 | Temp_Inf_L | LN | 0.113 | / |
| 33 | -57 | -33 | -21 | Temp_Inf_L | LN | 0.425 | 0.404 |
| 36 | -9 | -73 | 38 | Precuneus_L | CEN | / | 0.432 |
| 37 | -4 | -26 | 33 | Cingulum_Mid_L | CEN | 0.098 | / |
| 42 | -35 | 21 | -11 | Insula_L | DMN | 0.361 | 1.432 |
| 47 | -41 | 14 | 48 | Frontal_Mid_L | DMN | 0.112 | 0.989 |
| 48 | -25 | 20 | 51 | Frontal_Mid_L | DMN | 0.305 | 1.256 |
| 50 | -6 | -53 | 33 | Cingulum_Post_L | DMN | 0.083 | / |
| 51 | 32 | -31 | -22 | Fusiform_R | VN | 0.193 | / |
| 52 | 27 | -66 | -12 | Fusiform_R | VN | 0.373 | 4.76 |
| 58 | 13 | -86 | 29 | Cuneus_R | VN | 0.417 | 1.315 |
| 59 | 53 | -16 | 7 | Temp_Sup_R | SMN | 0.087 | / |
| 63 | 47 | -11 | 48 | Precentral_R | SMN | 0.108 | 0.819 |
| 64 | 41 | -22 | 60 | Precentral_R | SMN | 1.157 | 0.606 |
| 65 | 30 | -37 | 64 | Postcentral_R | SMN | 0.14 | 0.211 |
| 66 | 6 | -26 | 70 | Paracentral_Lobule_R | SMN | / | 0.629 |
| 68 | 50 | -24 | 42 | Postcentral_R | DAN | / | 0.661 |
| 69 | 38 | -45 | 49 | Par_Inf_R | DAN | 0.541 | 0.836 |
| 70 | 27 | -67 | 51 | Par_Sup_R | DAN | 0.512 | 1.931 |
| 73 | 28 | -3 | 59 | Frontal_Sup_R | DAN | 0.328 | / |
| 76 | 40 | 8 | 1 | Insula_R | VAN | 0.129 | / |
| 77 | 11 | -31 | 45 | Cingulum_Mid_R | VAN | 0.304 | 1.571 |
| 78 | 7 | 6 | 52 | Supp_Motor_Area_R | VAN | 0.272 | 0.846 |
| 79 | 12 | 35 | -20 | Rectus_R | LN | 0.635 | 1.727 |
| 86 | 43 | 16 | 45 | Frontal_Mid_R | CEN | 0.362 | 0.213 |
| 88 | 6 | 28 | 30 | Cingulum_Ant_R | CEN | 0.086 | / |
| 89 | 9 | -66 | 43 | Precuneus_R | CEN | 0.1 | / |
| 91 | 62 | -23 | -19 | Temp_Inf_R | DMN | / | 0.186 |
| 92 | 51 | 7 | -18 | Temp_Pole_Mid_R | DMN | 1.146 | / |
| 96 | 7 | 48 | 1 | Frontal_Sup_Medial_R | DMN | 0.096 | / |
| 98 | 26 | 24 | 50 | Frontal_Sup_R | DMN | 0.301 | 0.418 |
| 99 | 12 | -54 | 14 | Calcarine_R | DMN | 0.996 | 0.601 |
| 106 | 15 | 21 | -1 | vCaudate_R | DMN | 1.152 | 0.632 |
| 109 | -19 | -1 | -8 | NucleusAccumbens_L | LN | / | 1.014 |
| 110 | 12 | 12 | -8 | NucleusAccumbens_R | LN | 1.007 | 1.37 |
| 111 | -21 | 12 | -1 | vmPutaman_L | VAN | 0.19 | / |
| 113 | -17 | -12 | 23 | dCaudate_L | CEN | 0.089 | / |
| 117 | -19 | -13 | -22 | HIP_rostral_L | DMN | 0.165 | / |
| 121 | -4 | -18 | 5 | THAL_mPF_L | DMN | 0.131 | / |
| 122 | 6 | -18 | 9 | THAL_mPF_R | DMN | / | 0.195 |
| 124 | 12 | -18 | -2 | THAL_mPM_R | VAN | 1.264 | / |
